# Research Progress on Biomimetic Nanomaterials for Electrochemical Glucose Sensors

**DOI:** 10.3390/biomimetics8020167

**Published:** 2023-04-20

**Authors:** Lili Chi, Chunmei Zhang, Xuanyu Wu, Xianghao Qian, Hao Sun, Mengru He, Chunxian Guo

**Affiliations:** 1 School of Materials Science and Engineering, Suzhou University of Science and Technology, Suzhou 215009, China; 2 School of Environmental Science and Engineering, Suzhou University of Science and Technology, Suzhou 215009, China

**Keywords:** biomimetic nanomaterials, electrochemical, glucose sensing, noninvasive

## Abstract

Diabetes has become a chronic disease that necessitates timely and accurate detection. Among various detection methods, electrochemical glucose sensors have attracted much attention because of low cost, real-time detection, and simple and easy operation. Nonenzymatic biomimetic nanomaterials are the vital part in electrochemical glucose sensors. This review article summarizes the methods to enhance the glucose sensing performance of noble metal, transition metal oxides, and carbon-based materials and introduces biomimetic nanomaterials used in noninvasive glucose detection in sweat, tear, urine, and saliva. Based on these, this review provides the foundation for noninvasive determination of trace glucose for diabetic patients in the future.

## 1. Introduction

In recent years, diabetes has become an epidemic with a high incidence [1]. The blood glucose level of normal people ranges from 4.4 to 6.6 mmol L^−1^. If blood glucose levels exceed this range, it can lead not only to diabetes, but also to more serious complications that can seriously damage a person’s health [2]. Based on the recent report of International Diabetes Federation (IDF) in 2021, over 537 million adults are suffering from diabetes [3]. World Health Organization (WHO) predicted that the number would rise to 643 million by 2030 without action [4]. In 2022, the first target was stated at the World Health Assembly (WHA) with the aim that 80% of people living with diabetes will be diagnosed by 2030 [4]. In such a case, developing rapid, sensitive, and reliable glucose sensors is imminent. In addition, glucose monitoring is the foundation and prerequisite for diabetes detection and therapy.

**Figure 1:**
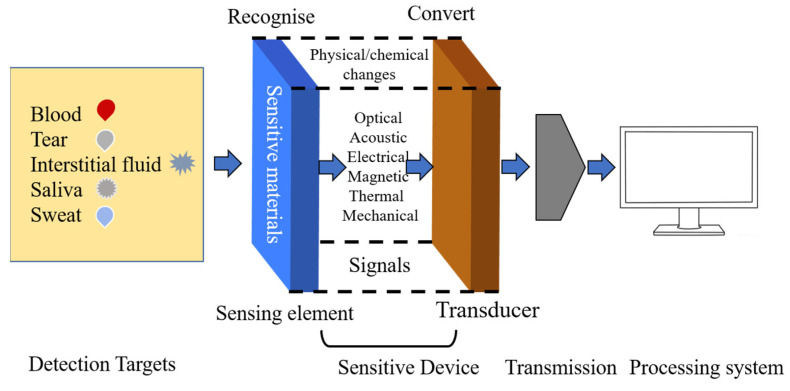
Structure and mechanism of glucose sensors.

Figure 1 illustrates the structure and mechanism of glucose sensor devices, which mainly contain four parts as follows: detection targets, sensitive device, transmission, and signal receiving system. Target molecules can be divided into two categories: invasive (blood [5]) and noninvasive (sweat [6,7,8], tear [9], saliva [10], and urine [11]). The traditional method of measuring glucose in blood by fingertip sampling not only brings psychological burden to patients, but also a cumbersome sampling process and strict operation. Using this method can carry risks of infection and inflammation. Therefore, it is necessary to develop wearable and implantable electrochemical glucose sensors. Sensitive materials can react with target molecules and produce physical and chemical changes, including optical, acoustic, electrical, magnetic, thermal, and mechanical signals. Based on different signals, various detection technologies have been developed in recent years, including spectroscopic methods, such as fluorescence, surface plasmon resonance [12], colorimetric analysis [13,14,15,16,17], and illuminating sensors [18,19,20,21]. However, these spectroscopic methods usually need complex instruments and professional personnels, which are not suitable for household use but scientific research institutions and hospitals. Compared with these methods, electrochemical methods have attracted much more attention because of their real-time detection, fast response, and simple and easy operation, which profit small-sized residential continuous monitoring and popularizing application of glucose detection in the future.

The key core part in electrochemical sensors for accurate detection of glucose is the sensitive materials used for sensing glucose, including biological materials and biomimetic materials [22,23]. Biological materials mainly include glucose oxidase (GOx) or glucose dehydrogenases (GDHs) [24,25]. In 1967, Updike and Hick designed and produced the first glucose oxidase electrode sensor, which marks the beginning of the glucose oxidase sensor [26]. GOx can oxidize glucose to hydrogen peroxide (H_2_O_2_) and gluconic acid, which can be used as the parameters for calculating glucose content. GDHs need cofactors flavin adenine dinucleotide (FAD) and pyrroloquinoline quinone (PQQ) to unbind nicotinamide adenine dinucleotide (phosphate) (NAD(P)) [27]. Although biological enzymes can oxidate or reduce glucose directly with fast response, high response intensity, and strong specificity [28,29], their high price, complex self-assembly steps, and environmental instability are unfavorable for their recycle and wide applications [30]. Therefore, inspired by the high efficiency properties of natural enzymes, in recent years, researchers began to design and synthesize biomimetic nanomaterials with high activity according to the structural characteristics of biological nanomaterials to replace biological nanomaterials.

Till now, biomimetic nanomaterials have had different expressions containing non-enzyme nanomaterials, enzyme-free nanomaterials, nanozymes [31], biomimetic nanozyme, and enzyme-mimicking nanomaterials [32]. In addition, biomimetic or non-enzyme nanomaterials become the fourth-generation glucose sensing materials, which have attracted increasing interest in recent years [33]. Compared with enzymes, biomimetic materials have a large specific surface area, good biocompatibility, stable structural properties, and easy-to-control chemical and physical properties [34,35,36].

Recently, reviews have mainly focused on two major aspects for electrochemical glucose sensors: new electrochemical detection technologies based on different targets [25,37,38] and biomimetic nanomaterials [33,39,40,41,42,43]. For various targets, Su’s group overviewed the implantable and wearable electrochemical glucose sensors, which contain saliva, sweat, tears, and interstitial fluid sensors [37]; Peng et al. discussed the mechanisms of electrochemical and optical sensors for glucose detection in body fluids, such as blood, saliva, sweat, interstitial fluid, tears, and urine [38]. For biomimetic materials, Chen’s group reviewed graphene-based materials for electrochemical glucose sensor [39]; He et al. overviewed noble-metal-based materials for electrochemical nonenzymatic glucose detection [33]; Akter et al. reported nanostructured nickel-based materials for nonenzymatic electrochemical glucose sensors [41]; and Wei et al. summarized electrochemical nonenzymatic glucose sensing materials, including noble metals, metal alloys, transition metals, metal oxides, and other materials [40]. However, there are few reviews about biomimetic nanomaterials focusing on improving electrochemical glucose sensing performance.

**Figure 2:**
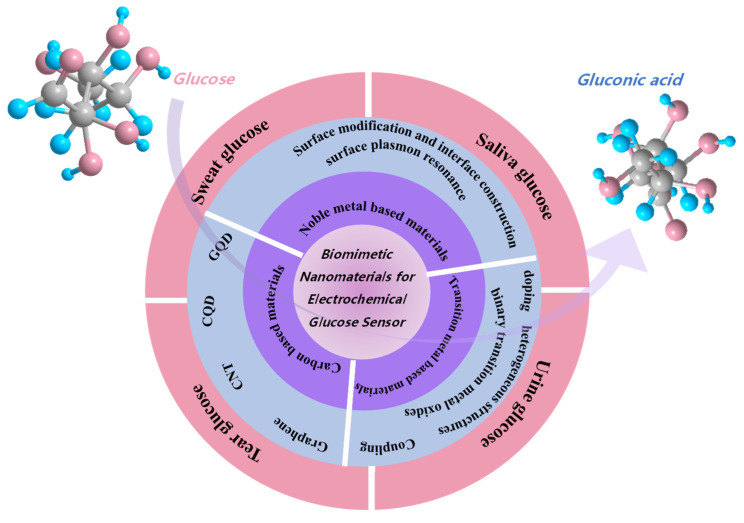
Illustration of this review for biomimetic nanoparticles for glucose sensors.

This review introduces biomimetic nanomaterials containing the methods to improve their glucose sensing properties and noninvasive nonenzymatic glucose detection in sweat, tears, urine, and saliva, as seen in Figure 2. Hence, the discussion and concepts presented in this review will be helpful for nonenzymic glucose detection in the future.

## 2. Biomimetic Nanomaterials

All the time, as three typical biomimetic nanomaterials, noble metal, transition metal oxides, and carbon-based materials have received extensive attention. Noble-metal-based nanomaterials have special optical properties [44], good chemical stability [45], controllable enzyme-like activity [46], and favorable biocompatibility [47] and are widely applied in industrial catalysis [48] and medical fields [49]. In 2007, Yan’s research group discovered for the first time that magnetic nanoparticles of ferric oxide have catalytic activity [50]. Later, the research on biomimetic nanomaterials based on transition metal oxides also increased rapidly. With the development of sensors, there is an increasing demand for wearable glucose sensors. Based on the excellent properties of the above three typical biomimetic nanomaterials, the methods to enhance the electrochemical glucose sensing performance of different biomimetic nanomaterials will be introduced in this review.

### 2.1. Noble-Metal-Based Biomimetic Nanomaterials

Noble-metal-based nanoparticles include noble metals and their compounds. Noble metal (Pt, Pd, Ir, Rh, Ru, and Au)-based materials have ideal adsorption properties, and these metals are often prepared as nanoparticles to increase their surface area and improve defect density [51,52]. It is generally believed that the interfacial properties of noble metal nanoparticles have great influence on their catalytic performance [53,54]. Such surface interface states include coating a layer of noble metal on the surface of other porous materials through chemical bonding or physical action, coating noble metal nanoparticles with hollow structure, sandwich structure, or surface plasmon resonance enhancement to improve the sensing performance of active materials.

The modification strategies of nanocatalysts are summarized in terms of surface modification and interface construction. In the end, the prospects and challenges of enzyme-free electrochemical glucose sensor nanocatalysts are presented. In this review, we will provide useful guidance for the further research on the catalytic oxidation of glucose by nanomaterials in the field of nonenzymatic glucose sensors.

**Figure 3:**
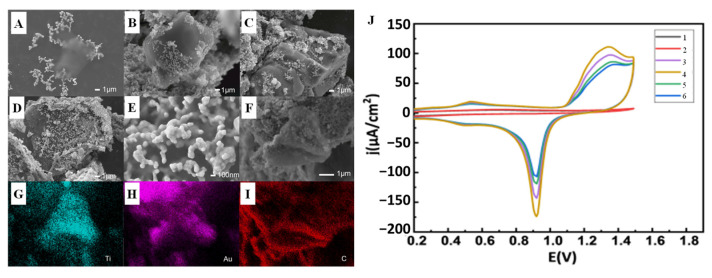
SEM images of the Au/MXene composite nanoparticles prepared by different amounts of MXene suspension: (**A**) 1 µL (**B**) 2.5 µL, (**C**) 5 µL, and (**D**) 10 µL; (**E**) SEM image of the porous foam structure of the Au nanoparticles on the surface of the MXene; (**F**–**I**) SEM and corresponding elemental mapping images of the Au/MXene composite nanoparticles; (**J**) CV scans of 1–6: GCE, MXene, and modified with different amount of Au/MXene/Nafion [55] with permission (Copyright © 2022, Chinese Physical Society).

Electrocatalytic reactions occur on the surface of catalysts. Therefore, increasing the number of active sites on the surface of biomimetic nanomaterials is the key to enhance the contact between electrolyte and catalyst. Bi et al. [55] prepared MXene by mild etching method and combined porous foam of Au NPs with MXene (Figure 3A–I) by in situ synthesis method. By controlling the mass of MXene during preparation, porous foams containing Au NPs were obtained for use in novel electrochemical nonenzymatic glucose sensors (Figure 3J). Au NPs adhere to porous foam, such as coral, on the surface of MXene by van der Waals force. This nonplanar structure will enhance the electrochemical reaction signal by increasing the exposed area and allowing more active sites for noble metal nanoparticles.

**Figure 4:**
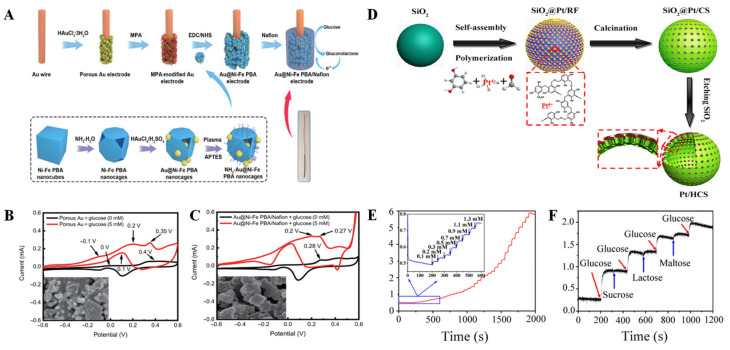
(**A**) Preparation of the Au@Ni-Fe PBA nanocages; CV responses of the porous Au (**B**) and Au@Ni-Fe PBA/Nafion (**C**) with and without glucose, and the insets show the surface morphology of materials [56] with permission (Copyright © 2022, Springer); (**D**) Schematic illustration of the formation of Pt/HCS; (**E**) Amperometric response of Pt/HCS to successive addition of glucose at the potential of 0.6 V in N_2_-saturated 0.1 M PBS solution (pH = 7.4); (**F**) the current response of Pt/HCS to different analytes (sucrose, lactose, and maltose) [57] with permission (Copyright © 2018, American Chemical Society).

Nanometal powders are coated and modified by physical, chemical, and other methods by changing the surface composition of nanometal powders. Structure and state not only can improve the dispersion of nanometal powder, improve the compatibility between nanometal powder and other substances, but also can produce new physicochemical properties and new functions, as well as reduce production costs. Therefore, as an important functional material, nanometal cladding materials have a wide range of application prospects. Shen et al. [56] first etched Prussian blue analogs (PBA) nanocubes, and then obtained AuNP-PBA by inlaying gold nanoparticles (Au NPs) into the etching cavity by in situ reduction of HAuCl_4_. This suitable structure (Figure 4A) promotes a tight connection between Au NPs and Ni-Fe PBA nanocages, enabling efficient electron transport, increased specific surface area, enhanced conductivity, and sensing performance. Electrochemical testing shows that AuNP-PBA sensor has wide linear detection range (0.01~16 mM), good anti-interference performance, and fast response speed. What is more, more importantly, the sensor is reusable (Figure 4B,C).

In 2018, we also conducted studies on the preparation of Pt/HCS by using hollow carbon spheres (HCS)-confined Pt nanoparticles [57]. The large surface area and porous structure of HCS make them an attractive carbon carrier to confine bare platinum nanoparticles with an average particle size of about 3.13 nm into mesoporous shells of HCS (Pt/HCS) (Figure 4D). Pt/HCS have a larger surface area (566.30 m^2^ g^−1^) than HCS, which is due to the deposition of Pt nanoparticles. The synthetic Pt/HCS were highly selective as nonenzymatic materials for the detection of glucose in neutral solution (Figure 4E,F).

**Figure 5:**
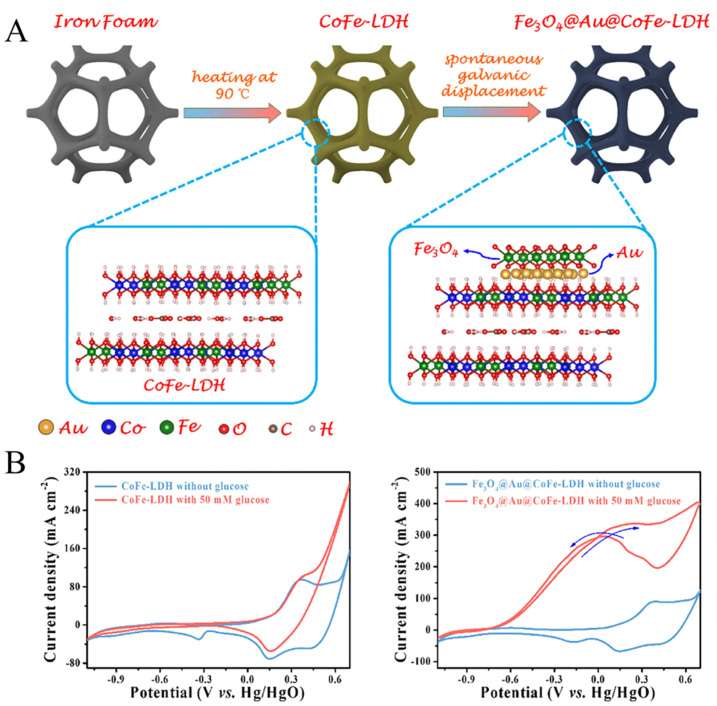
(**A**) Design and synthesis of Fe_3_O_4_@Au@CoFe-LDH. (**B**) CV curves of CoFe-LDH and Fe_3_O_4_@Au@CoFe-LDH with and without 50 mM glucose [58] with permission (Copyright © 2022, Elsevier).

The formation of good designed interfaces between two or more metal components that are more conducive to producing synergistic effects on the nanoscale contributes to significantly improving the activity and stability of glucose catalysts. The constraint effect of mezzanine and sandwich structure also leads to higher long-period stability. The reasonably designed sandwich structure promotes a new concept for the design of high-sensitivity and highly stable nonenzymatic glucose electrodes. For example, it has been reported that Sun et al. [58] successfully synthesized Fe_3_O_4_@Au@CoFe-LDH electrocatalyst, where Au was confined between Fe_3_O_4_ and CoFe-LDH to form a triplet structure (Figure 5A). Layered dihydroxides (LDH) have the advantages of large surface area and regulable structure and composition. More importantly, LDH transition metal elements in low- and medium-value states will be conducive to the formation of Au. According to previous reports, increasing Au dispersion on the LDH can significantly enhance the detection sensitivity [59,60]. In addition, the size of gold nanoparticles also affects the detection results [8]. The Fe_3_O_4_@Au@CoFe-LDH glucose sensor shows good electrochemical response (Figure 5B).

When the light incident on nanoparticles composed of precious metals, if the frequency of the photo incident is matched with the overall vibration frequency of the electrons conducted by the precious metal nanoparticles, the nanoparticles will have a strong absorption effect on the photon energy, which will lead to the resonance of the local surface plasma. Au, Ag, and other noble metal nanoparticles have a strong effect of local surface plasmon resonance and show strong spectral absorption, so that the local surface plasmon resonance spectrum can be obtained. Because the absorption wavelength at the peak of the absorption spectrum is affected by the microstructure characteristics of the material, such as composition, shape, structure, size, and local electrical conductivity, therefore, we can study the microscopic composition of nanoparticles by analyzing local surface plasmon resonance spectra. Meanwhile, based on the principle that LSPR absorption spectrum is extremely sensitive to the surrounding medium, chemical sensors and biosensors based on optical signals can be developed.

**Figure 6:**
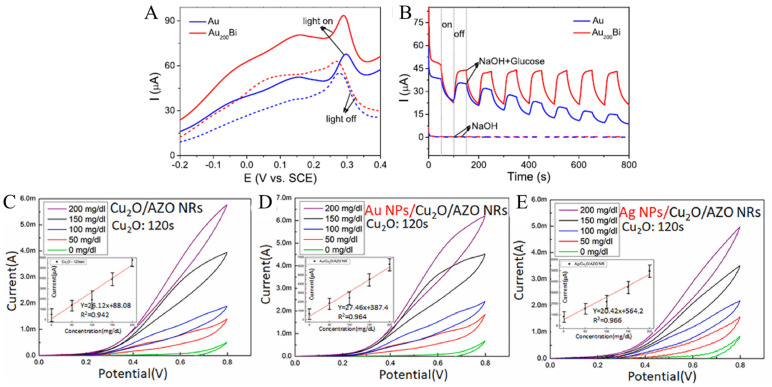
(**A**) Linear sweep voltammetry (LSV) curves of glucose oxidation with and without illumination; (**B**) photocurrent responses of Au and Au_200_Bi with and without 1 mM glucose at 0.15 V [61] with permission (Copyright © 2022, American Chemical Society). The CV curves of Cu_2_O/AZO NRs (**C**), Au NPs/Cu_2_O/AZO NRs (**D**), and Ag NPs/Cu_2_O/AZO NRs (**E**) [62] with permission (Copyright © 2022, Elsevier).

Zhu’s group [61] synthesized an ultra-low-content bismuth anchor aerogel with plasma-element properties to enhance nonenzymatic electrochemical glucose sensing. Thanks to the unique structure of the aerogel and the synergistic effect of Au and Bi, the optimized Au_200_Bi aerogel significantly improved the glucose oxidation activity compared with Au aerogel. The bimetallic Au_200_Bi aerogel with a wider light dependence showed further plasma promoting glucose electrooxidation activity under plasmon resonance excitation. When the Au_200_Bi and Au electrodes were irradiated by a light source, a further significant enhancement of the current was observed (Figure 6A). In particular, the activity of Au_200_Bi aerogel at 0.15 V potential is 1.8 times higher than that of Au aerogel under plasmon excitation. This result can also be verified by the photocurrent response of Au and Au_200_Bi aerogel at a constant potential of 150 mV (Figure 6B). Due to the improved performance, a nonenzymatic glucose electrochemical biosensor was constructed to detect glucose with high sensitivity. This kind of plasma element can promote the electrocatalytic activity of bimetallic aerogel through co-operative strategy and has potential application in various research fields.

Chen et al. [62] used surface plasmon resonance of Au and Ag nanoparticles (NPs) to stabilize hybrid Cuprous oxide (Cu_2_O)/aluminum-doped zinc oxide nanorods (AZO NRs) for nonenzymatic glucose sensors. The surface plasmon resonance effect of Au NPs is clearly shown in Figure 6D compared with the cyclic voltammetry (CV) of Cu_2_O/AZO NRs (Figure 6C). This effect causes the occurrence of electrocatalytic activity, so that the oxidation current changes dramatically in the forward scanning (towards high potential) phase. At the higher concentrations of 100–200 g L^−1^, the large oxidation current (Ipa) and redox current difference (Ipa-Ipc) were more significant. Additionally, in Figure 6E, CV changes prove the function of Ag NPs on surface plasmon resonance, which results in a steady rise in current at varying concentrations of 0 to 200 g L^−1^.

In recent years, the research of precious-metal-based bionic catalyst has made a lot of research results and progress, especially in the optimization of structure and composition control. However, due to the high cost, noble-metal-based catalysts cannot be prepared on a large scale, which hinders their further development in the field of non-enzyme electrochemical glucose sensors. More importantly, the kinetic evolution of noble-metal-based glucose catalysts has not been clearly elucidated, which is a key scientific question to reveal the behavior and mechanism of glucose oxidation. In conclusion, it is still necessary to fully develop diversified noble metal nanomaterials and catalysts with low noble metal content and devote to mechanism research to provide more options and guidance for comprehensive engineering research.

### 2.2. Transition-Metal-Based Biomimetic Nanomaterials

Nonvaluable transition metals and their oxides have been widely used in the preparation of efficient enzyme-free glucose sensors [63]. These metals, located in the third row of the periodic table, are relatively inexpensive and can react quickly and sensitively to glucose molecules [64]. Here, we will discuss in depth biomimetic material oxides based on Mn, Fe, Co, Ni, and Cu and methods to improve the performance of their nonenzymatic electrochemical glucose sensors. There are mainly heterogeneous structures, doping, coating, formation binary transition metal oxides, etc.

**Figure 7:**
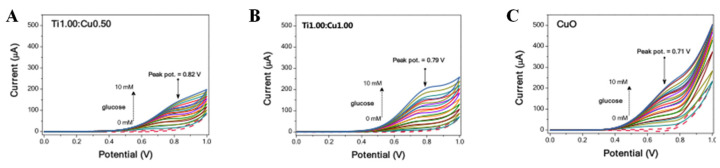
CV curves of materials in the presence of glucose from 0 to 10 mM: (**A**) Ti1.00:Cu0.50, (**B**) Ti1.00:Cu1.00, and (**C**) CuO [67] with permission (Copyright © 2020, Royal Society of Chemistry).

The synergistic effect of metal–metal oxide heterostructures has been proved to enhance electrocatalytic performance [65,66]. Metal–metal oxides produce metal-supported interfaces and strongly coupled interactions that not only facilitate catalyst stability, but also accelerate electronic conductivity. Tobaldi et al. [67] studied the photochemical properties of CuO–TiO_2_ heterojunctions used for glucose sensing in alkaline media. In the CuO–TiO_2_ heterojunction structure, TiO_2_ and Cu-based nanoparticles are strongly interwoven, and the size of CuO–TiO_2_ is smaller than pure CuO, which exposes more active sites for glucose electrooxidation (Figure 7). The improved photochemical properties in CuO–TiO_2_ heterojunctions may be due to a synergistic effect between the microstructure characteristics and the efficient separation of photoexcitons generated at the heterojunction. In conclusion, CuO–TiO_2_ heterojunction can effectively promote glucose oxidation in alkaline medium. Qi et al. [68] reported an Fe-doped induced crystalline/amorphous NiCo_2_O_4_ core/shell heterostructure used for highly sensitive detection of glucose. The existence of thin amorphous shell can efficiently accelerate the electron transfer and expose more effective active sites. This unique nucleated/amorphous shell structure of NiCo_2_O_4_ shows potential as an effective electrocatalyst for glucose sensing.

**Figure 8:**
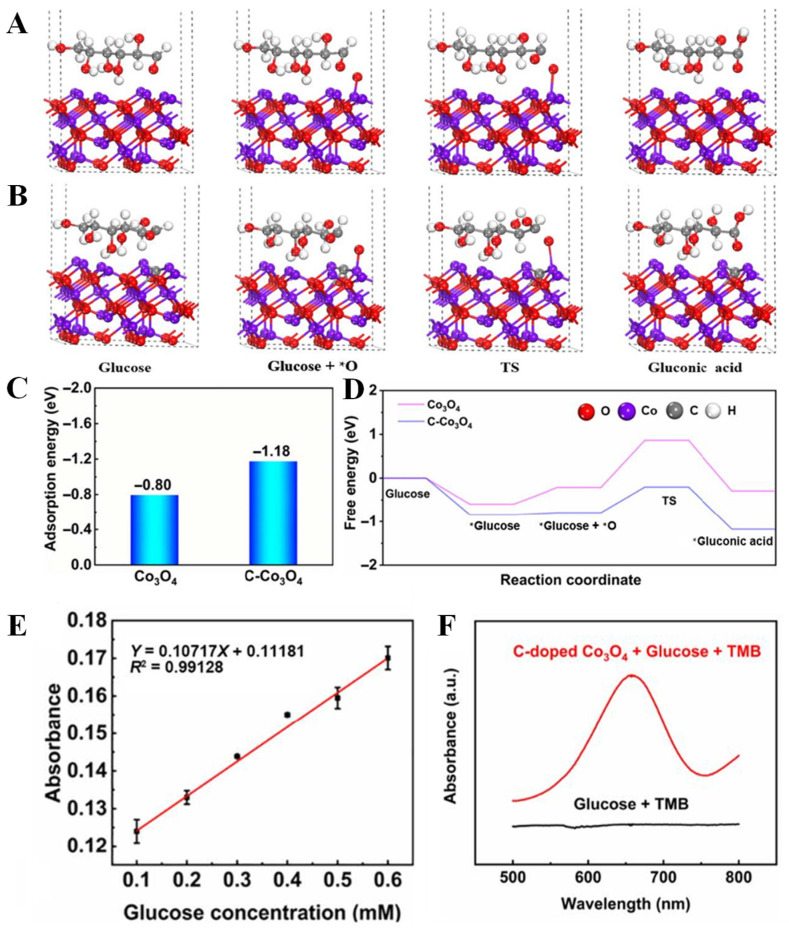
DFT calculations of glucose reaction pathway over (**A**) Co_3_O_4_ and (**B**) C-doped Co_3_O_4_; (**C**) the adsorption energy of adsorbed glucose; (**D**) free energy step of different reaction pathways. (**E**) Linear range of C-doped Co_3_O_4_; (**F**) UV detection glucose [32] with permission (Copyright © 2022, Springer).

**Figure 9:**
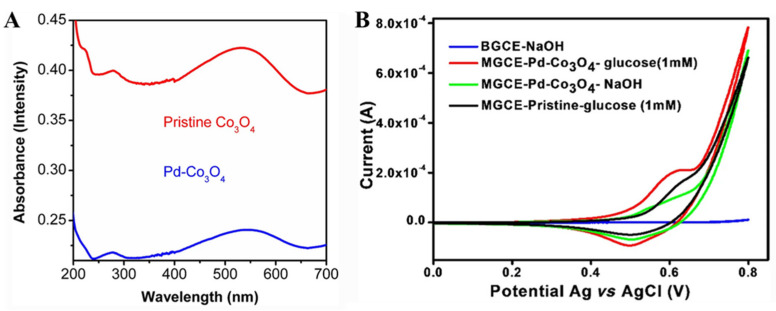
(**A**) UV-visible spectra of pristine Co_3_O_4_ and Pd-Co_3_O_4_ nanostructures. (**B**) CV curves of BGCE and MGCE with Pd-Co_3_O_4_ with and without glucose [70] with permission (Copyright © 2022, Indian Academy of Sciences).

What is more, it is an effective strategy to improve the performance of transition metal oxides by doping nonmetallic C, because nonmetallic C can change the energy band structure of transition metal oxides and improve their inherent properties. Therefore, C-doped transition metal oxides become a potential nanoartificial enzyme. Kang et al. [32] designed a strategy to obtain high-performance nanozymes by supercritical CO_2_ fluid technology. In this strategy, C-doped Co_3_O_4_ (C-Co_3_O_4_) nanozymes were prepared by one-step calcination process using poly-(methyl vinyl ether-co-maleic anhydride) and Co(NO_3_)_2_ as raw materials. By density functional theory (DFT) calculation, it was found that the catalytic site of C-Co_3_O_4_ showed a unique electronic structure (Figure 8A–D), which changed the surface of the material, so that more electrons fill the antibond between the two molecular orbitals, significantly improving the performance of the glucose sensor. The performance of the sensor is also related to the amount of doping material (Figure 8E,F). The combination of precious metals and transition metal oxides (TMOS) can effectively expand their applications in electrochemistry [69]. Studies have shown that Pd nanoparticles have good properties, such as significant catalytic activity and electrical conductivity, and also are used together with Co_3_O_4_ as a composite material for glucose detection. Chang et al. [70] successfully prepared Pd nanoparticles on Co_3_O_4_ nanostructures by UV reduction method under alkaline conditions for the first time, which was used for sensitive enzyme-free glucose sensor. The Co_3_O_4_ nanostructure modified by Pd nanoparticles has good electrochemical activity and can be used for the determination of the selectivity and sensitivity of glucose (Figure 9). Yang et al. [69] constructed Cu_3_Pt/Cu_2_O nanorods array on copper substrate by a simple method and used it as a nonenzymatic glucose detector. Cu_3_Pt nanorods can accelerate electron transfer. When used as a glucose detector, the Cu_3_Pt/Cu_2_O nanorod array provides enhanced linear detection range, high response sensitivity, fast response time, low detection limit, and high selectivity. Naik et al. [71] reported the simple synthesis of NiCo_2_O_4_ (NCO) and NiCo_2_O_4_-Pd (NCO-Pd) nanosheets by electrodeposition. Compared with naked NCO nanosheets, the glucose sensing performance of NCO-Pd nanosheets is enhanced. It is also confirmed that Pd-doped NiCo_2_O_4_ has more charge transfer, indicating that Pd-doped NiCo_2_O_4_ has superior charge transfer kinetics, which supports higher glucose sensing performance.

In order to pursue high efficiency and stability, researchers have been using different methods to change the structure and electronic properties of the active site. A common strategy is to change the electronic structure of a metal by forming a bimetallic structure, different metals and combinations have been explored, with binary metal oxides, such as Hou et al. [72] constructed multi-valent copper-based oxide composite nanofibers (Cu_x_O-CNFs). Compared with TiO_2_/CuO CNF, the sensitivity of TiO_2_/Cu_2_O/CuO CNF to glucose sensor (0–2 mM) can be increased. The enhanced sensitivity contributes to the TiO_2_ content in the original TiO_2_/CuO CNFs. Therefore, in the actual experiment process, the proportion of raw materials is one of the key factors to be solved in our regulation of material properties. In addition, spinel are relatively mature nanomaterials developed for biomimetic catalysis of glucose. Seong et al. [73] proposed an effective nitrogen doping strategy to synthesize the oxygen vacancy of Ni-Co oxide (N-Ov/NiCo_2_O_4_-350) nanowire arrays. The modified electrode has obvious nanoporous structure and favorable electronic structure, thus significantly increasing the specific surface area and suitable electron/ion diffusion network. Meanwhile, the nonenzymatic glucose sensor with N-Ov/NiCo_2_O_4_-350 achieves a wide linear detection range and ultra-high sensitivity, the response time is short, about 2.2 s, and the detection limit is low, 20 nM (S/N = 3). The great glucose sensing capabilities of N-Ov/NiCo_2_O_4_-350 hybrid nanostructures demonstrate the potential of electrodes. Liu et al. [74] synthesized the binary metal oxide CuCo_2_O_4_@NiCo_2_O_4_ and studied its catalytic ability for glucose. The two independent components that make up this hybrid electrode both have good electrical conductivity and excellent catalytic properties for glucose, so the combination of the two active materials can provide more catalytic sites for glucose oxidation. Experiments also prove that CuCo_2_O_4_@NiCo_2_O_4_ has excellent glucose sensing performance, including ultra-high sensitivity, fast response time, a wide linear range, and acceptable detection limits.

**Figure 10:**
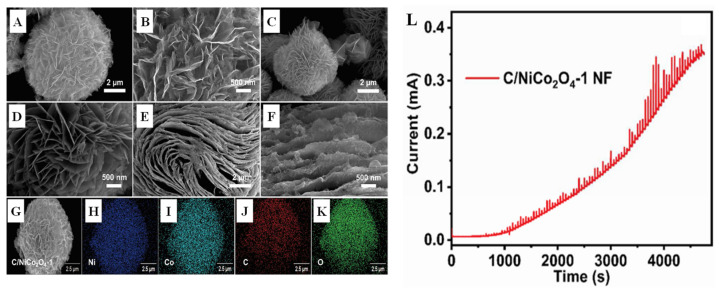
SEM images of (**A**,**B**) NiCo_2_O_4_ follower, (**C**,**D**) C/NiCo_2_O_4_-1 follower, and (**E**,**F**) C/NiCo_2_O_4_-2 flake; (**G**–**K**) the elemental mapping of C/NiCo_2_O_4_-1; (**L**) the amperometry profile of C/NiCo_2_O_4_-1 NF@GCE with a low to high concentration of glucose from 0.0001 to 15.28 mM [75] with permission (Copyright © 2022, Elsevier).

Shape and structure also affect material properties. Sivakumar et al. [75] synthesized Co_2_O_4_-NF with flower-like structure by a simple hydrothermal method for nonenzymatic glucose sensing (Figure 10). A floral structure of C/NiCo_2_O_4_-1 NF material covers a carbon surface. C/NiCo_2_O_4_-1 NF showed remarkable electrochemical properties for glucose oxidation. Guo et al. [76] systematically synthesized a uniform NiCo_2_O_4_ nanowire array on a flexible carbon cloth (CC). Then, the ZIF-67 nanocubes were grown in situ on the prepared NiCo_2_O_4_ nanowires to form the mixed nanostructures. This Co_3_O_4_/NiCo_2_O_4_/CC electrode has excellent glucose sensing properties, including extremely high sensitivity, wide linear range, low detection limit, and fast response time. Feng et al. [77] constructed a nonenzymatic glucose sensor by using NiCo_2_O_4_ hollow nanocages (NiCo_2_O_4_-HNCs) derived from the cobalt-based zeolite imidazole framework (ZIF-67) as a catalyst. As a key component of glucose sensor, NiCo_2_O_4_-HNCs-modified glassy carbon electrode (NiCo_2_O_4_HNCs/GCE) shows high electrochemical catalytic activity for the oxidation of glucose in alkaline medium.

Unlike precious metals, such as Pt, these transition metal materials, which include Mn, Fe, Co, Ni, and Cu, are inexpensive and their resources are also abundant on Earth. This advantage does not limit their large-scale use in practical applications. Therefore, the application of transition metal-oxide-based nano-biomimetic materials in sensors is very promising. However, transition-metal-based nanomaterials also have some shortcomings, such as the preparation process usually needs to meet certain temperature requirements, strict reaction conditions, long reaction time, the catalytic glucose reaction site is limited, and the analysis of the sensing mechanism is difficult, which still needs further research. In addition, transition-metal-based biomimetic nanomaterials exhibit glucose active sensing performance in alkaline solutions in most cases. In other words, the glucose sensing performance of this kind of material is greatly affected by pH. Therefore, active materials in neutral solutions need to be further studied.

### 2.3. Carbon-Based Biomimetic Nanomaterials

With the development of nanotechnology, the research of carbon-based nanomaterials (CNs) has entered the frontier stage from macro to nano. Carbon has different allotropes, such as carbon nanotubes (CNTs), carbon quantum dots (CQDs), graphene and its derivatives, etc. Compared with other nanomaterials for electrochemical applications, carbon has excellent physicochemical properties, such as chemical inertness, large surface area, wide potential window, good biocompatibility, unique electronic properties, better electrocatalytic activity, and easy functionalization. Recently, CNs have been widely used in the field of sensing [78]. Carbon points (CDs) are typically quasi-spherical nanoparticles smaller than 10 nm that exhibit good electrocatalytic activity, good conductivity, water solubility, and further functionalization with a variety of biological, organic, inorganic, or polymers. There have been a lot of studies on the application of CDs in electrochemical sensors and biosensors [79]. Carbon nanofibers (CNFs) are similar to carbon nanotubes in structure and properties. CNFs have good thermal conductivity, excellent electrical conductivity, high porosity, and high specific surface area and are considered strong substrates for non-enzyme biosensors due to their high electrical conductivity and high specific surface area [80].

CNTs are representative and have excellent functions among nanomaterials. The theoretical tensile strength of carbon nanotubes is 100 times that of steel, while the density is only one sixth that of steel. CNTs can be used as plate materials for double-layer supercapacitors to achieve very high specific power. Because carbon nanotubes have many attractive electronic properties, they have a wide range of applications in radio communications, hydrogen storage batteries, aerospace, military, and other fields [81]. In fact, carbon nanotubes are widely used in the manufacture of electrochemical sensors due to their large surface area ratio, excellent electrical conductivity and good stability. In addition, carbon nanotubes in nanocomposites can facilitate electron transport and space diffusion of electroactive sites [82].

Methods to improve the properties of carbon nanotube materials are often combined with other organic or inorganic materials in the practical process. For example, Muqaddas et al. [83] developed a fibrous microelectrode consisting of copper-oxide-modified carbon nanotubes (CuO@CNTFs) as a flexible wearable glucose sensor with significant catalytic activity. The fiber microelectrode (CuO@CNTFs) has been used for glucose sensing, with a sensitivity of ~3000 μA mM^−1^ cm^2^, a minimum detection limit of 1.4 μM, and a linear range of 13 mM. The excellent performance of the microelectrode is attributed to the synergistic effect between the electrocatalytic activity of CuO nanoparticles and the excellent conductivity of carbon nanotube fibers. Vinoth et al. [84] prepared glucose sensors by fixing zinc oxide quantum dots (ZnO QDs) onto multiwall carbon nanotubes (MWCNTs) nanocomposites. The synthesized nanocomposites were used as electrochemical detection sensors for glucose.

CQDs and graphene quantum dots (GQD) are 0 D materials with good electrical conductivity, adjustable fluorescence properties, low toxicity, small size, and easy modification, so they are widely used in the field of sensing and detection. Wu et al. [85] prepared copper and nitrogen co-doped CQDs (Cu, N-CQDs) by a simple hydrothermal method and used them as a new sensing material to manufacture sensitive nonenzymatic glucose sensors. It is noteworthy that the introduction of Cu into the prepared materials results in a fourfold increase in glucose response compared to that of N-CQD. The sensor has excellent sensitivity and selectivity in the detection of glucose in alkaline media, with a linear range of 5–700 mM, and the detection limit is 1.22 mM (S/N = 3). In addition, the prepared sensors were used to detect glucose in fermented samples, suggesting that Cu, N-CQDs are promising candidates for sensitive glucose detection. Maaoui et al. [86] also prepared carbon quantum dots modified with copper oxide nanostructures (CQDs/Cu_2_O) and evaluated their potential in electrochemical nonenzymatic glucose sensing. The sensor shows excellent electrocatalytic performance for glucose oxidation in alkaline solution. Kipnusu et al. [87] prepared nitrogen-boron-doped CQDs. Incorporating CQDs into transparent nanoporous silica (pSiO_2_) films (thickness 50 µm) forms CQDs-pSiO_2_ composites. CQDs-pSiO_2_ is also sensitive to glucose as low as 1.0 g L^−1^ up to 100 g L^−1^.

GQDs are excellent nanocarbon materials for glucose sensing due to their low toxicity, excellent solubility, high electron transfer ability, and stability [88,89]. Heteroatomic doping, especially N doping, can alter the surface state of GQDs, provide more active sites for GQDs, and give them new properties. Wu et al. [88] prepared NH_2_-GQDs doped with NiCo_2_O_4_ grown on carbon cloth by hydrothermal method. Because of the high electron transfer rate and synergistic effect, NH_2_-GQDs/NiCo_2_O_4_ significantly improves its electrochemical performance. The glucose sensor shows excellent selectivity and reproducibility. Precious metal control of quantum dots is also a feasible strategy. Lima et al. [89] modified the surface of a carbon composite electrode with gold nanoparticles conjugated with GQDs (Au@GQDs) for sensitive nonenzymatic glucose detecting. The characterization of the modified electrochemical sensor revealed the rough hydrophilic surface of the composite material, the high dispersion of graphite in the polymer matrix, and the successful modification of spherical surface Au@GQDs nanoparticles. The graphite/Au@GQDs was used for the glucose sensing of synthetic saliva samples.

Graphene is a well-known carbon material with a honeycomb lattice and ultrathin sheets of single-atom thickness. Graphene-based materials have broad application prospects in electrochemical glucose sensing. Compared with GO and reduced GO, raw graphene has less interfacial contact; therefore, GO and reduced GO are widely used in high-performance glucose sensors [42]. Zhang et al. [18] reviewed the preparation status of graphene-based electrochemical glucose sensing materials. G. nana Kumar reviewed nonenzymatic electrochemical glucose sensors based on graphene [18]. Using flower-like gold nanostructures (F-Au NTs) and graphene oxide (GO) as substrates. Asen et al. [90] electrodeposited F-AuNTs on GO nanosheets to synthesize F-Aunts-Go complexes. F-Au NTs-GO/SPE showed good performance for glucose detection, with linear range of 0.16–82 μM and 0.16–5 mM, high sensitivity of 474,617 μA mM^−1^ cm^−1^, and low detection limit of 123 μM. Li et al. [91] synthesized PtNi alloy nanoparticles (PtNi alloy graphene) uniformly dispersed on graphene as an efficient electrode material for glucose detection. Based on the modified PtNi alloygraphene/GC electrodes, it is found that the graphene/GC electrodes of PtNi alloy exhibit excellent electrocatalytic properties for glucose oxidation. Wang et al. [92] prepared a unique Ni/NiO hybrid nanoparticle structure that has mixed valence states, is coated with nitrogen-doped graphene, and is cross-linked and developed its remarkable characteristics in efficient nonenzymatic glucose sensing. Nitrogen-doped graphene (NG) can act as a highway for electron transport due to its remarkable electrical conductivity. Second, the graphene shell can inhibit the agglomeration of Ni/NiO particles, thereby improving structural stability and long-term performance in electrochemical processes. Although rGO exhibits excellent electrical conductivity, high mechanical strength, and large surface area, it is insoluble and difficult to disperse in solvents due to its high hydrophobicity and strong van der Waals interactions [93,94]. On the other hand, GO provides abundant oxygen-containing functional groups on the surface, providing hydrophilicity and high negative charge density [95], which can effectively bind heavy metal ions to form metal complexes on the surface of GO through strong electrostatic interactions and the co-ordination of metal ions with oxygen-containing functional groups. Therefore, it is proposed to use the adsorption property of GO to adsorb Cu (II) ions. Phetsang et al. [96] used copper (II)/rGO-modified screen-printed carbon electrodes for nonenzymatic glucose sensors with high sensitivity and low cost. The proposed sensor has good electrocatalytic activity for glucose oxidation. Copper (II)/rGO-based sensors have excellent performance and have great potential for quantifying glucose in real samples.

In summary, it is often possible to further exploit the efficiency and effectiveness of metal oxides by incorporating graphene into composites to achieve synergies [97]. Thus, enzyme-free glucose sensors can become more cost-effective while maintaining or even improving their performance and accuracy.

Carbon materials have been widely studied in glucose sensors because of their large surface area, high conductivity, and sensitive activity. However, it is worth noting that carbon materials lack of specificity. Therefore, it is necessary to design and synthesize the high selectivity carbon composite materials using carbon materials as substrate materials.

Most of the biomimetic noble metal, transition metal, and carbon-based nanomaterials described above exhibit high catalytic activity for glucose oxidation in alkaline media. In addition, direct analysis of glucose in a neutral environment is also a significant advantage of some nanomaterials in sensing glucose, considering biocompatibility, which is also listed in Table 1.

## 3. Biomimetic Nanomaterials for Noninvasive Electrochemical Glucose Sensors

The traditional method to detect diabetes is the detection of blood glucose concentration in serum. Although it is reliable, accurate, and used widely in hospitals, its time consumption is still a problem troubling people.

Developing a noninvasive, wearable glucose sensor is particularly urgent. Most studies have shown that glucose is widely present in skin surface liquids (such as sweat, tears, and so on) in the human body [98,99]. In recent years, many studies have adopted noninvasive methods to detect glucose content in the human body; at the same time, the emergence of the Internet of Things has enabled the development of smart wearable devices capable of real-time monitoring of relevant biomarkers in human body fluids, such as by detecting sweat [98], tears [100], urine [101], and saliva [102]. These wearable point-of-care devices avoid painful skin piercings and blood draws. We will introduce biomimetic nanomaterials based on the following four noninvasive glucose sensors, as illustrated in Table 2.

### 3.1. Biomimetic Nanomaterials for Sweat Glucose Sensors

Compared with blood, human sweat is easier to be collected. Sweat consists of many health-related signal molecules, such as lactate [120], glucose [121,122,123], uric acid [124], ascorbic acid [125], Na^+^, K^+^, etc. [126]. Among these molecules, glucose in sweat has good correlation with glucose in blood, which has been used in noninvasive electrochemical wearable sensors [7,103,122,127,128,129]. However, the normal level of sweat glucose is less than 0.2 mM, which is lower than that in blood serum, with the range of 5.6–6.9 mM. For patients with diabetes, the content range of glucose in sweat is 0.28–1.11 mM, which is in good correlation with blood glucose concentrations. Therefore, diabetes can be indirectly determined by measuring glucose content in sweat. It is necessary to design a reliable noninvasive monitoring glucose sensor for detecting diabetes. However, compared to commercial glucometers, the application of sweat glucose sensors is limited by their low stability and sensitivity [130]. In addition, since sweat glucose levels are 100 times lower than blood sugar levels, direct monitoring of sweat glucose remains a huge challenge and detection sensitivity is a key issue that must be addressed [131]. Due to the complex chemical environment of human sweat, high selectivity is essential for sensors. Although GOx has high selectivity and sensitivity, the application of the flexible sweat glucose sensors is still a great challenge. From first until now, enzyme-based glucose biosensors have the serious problem of low stability. Consequently, nonenzymatic sweat glucose sensing materials are vital for noninvasive wearable devices. Some biomimetic nanomaterials have been used in noninvasive wearable devices. The wearable devices usually include a flexible three-electrode system made by different flexible materials. Biomimetic nanomaterials used in electrochemical sweat glucose sensors are not abundant and they can be divided into three categories: MXene-based materials, metal oxides/layered double hydroxides/metal−organic frameworks (MOF), and cellulose-based functional materials.

**Figure 11:**
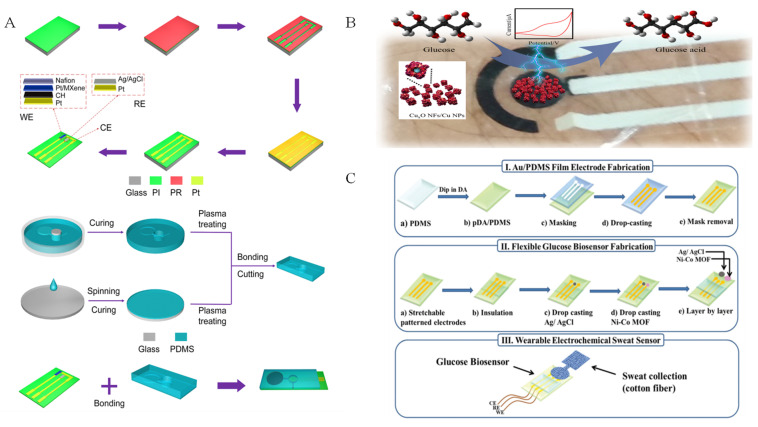
Electrochemical sweat glucose sensors based on different biomimetic nanomaterials: (**A**) Pt/MXene [104] with permission (Copyright © 2023, American Chemical Society); (**B**) Cu_2_O NFs/Cu NPs with permission (Copyright © 2023, MDPI); (**C**) Ni–Co MOF nanosheet coated Au/PDMS film [7] with permission (Copyright © 2022, The Royal Society of Chemistry).

MXene (Ti_3_C_2_T_x_) as a 2D transition metal carbon material has been used in wearable sensors because of its excellent conductivity, biocompatibility, and flexible tensile property [55,103,132]. As seen in Figure 11A, Li et al. [103] used Pt/MXene as active sensing materials for nonenzymic electrochemical glucose detection, and it exhibited a broad linear range of glucose detection (0−8 mM) under neutral conditions. In addition, with the protection of a conductive hydrogel, the stability of Pt/MXene glucose sensor was enhanced and could be used in a glucose sensor. Besides Pt/MXene, Au/MXene also exhibited a high sensitivity of 22.45 µA mM^−1^ cm^−1^ and a wide linear range of 1−12 mM [55].

Metal oxides are also used in glucose detection and applied to sweat glucose. For example, Kang et al. [104] designed and developed Cu_x_O nanosheets (NFs)/Cu nanoparticles (NPs) nanocomposites as sensitive materials for noninvasive sweat wearable glucose sensors, as shown in Figure 11B. Because Cu_x_O NFs confers more active sites, the prepared materials exhibited high sensitivity (779 μA mM^−1^ cm^−2^) for noninvasive wearable sweat sensing. This Cu_x_O NFs/Cu NPs-based sensor has a low detection limit (79.1 nM) and can detect changes in glucose levels in sweat during daily life. This method provides a simple method for the design of copper oxide nanomaterials for a noninvasive wearable glucose sensor.

Layered double hydroxides (LDH) biomimetic materials also have been investigated and used in electrochemical glucose sensing because of their simple preparation, adjustable chemical composition, and excellent oxidation reducibility [41,133]. Bimetallic LDH, especially Ni-based bimetallic LDH, have attracted much attention in glucose sensing. For example, NiAl LDH [134], NiFe LDH [135], NiCo LDH [133,136,137,138], and NiCu LDH [139] have been used in electrochemical glucose detection. However, few LDH biomimetic materials have been used in detecting glucose in sweat sensors, which will be probably a further research direction.

Different from bimetallic LDH biomimetic materials, bimetallic MOF materials are not only used for nonenzymatic glucose electrochemical detection, but also applied in sweat glucose sensors. As seen in Figure 11C, Sun et al. [7] prepared a wearable electrochemical sweat sensor using Ni-Co MOF as an active sensing biomimetic material modified on Au/polydimethylsioxane (PDMS). The sensor exhibited high sensitivity of 205.1 μA mM^−1^ cm^−2^, with a linear range of 20–790 μM. In addition, Shu et al. [105] fabricated Ni-Co MOF nanosheet as a sensitive material, which was coated on Ag/reduced graphene oxide/polyurethane (Ag/rGO/PU). Ni-Co MOF enhanced the fiber sweat glucose sensor with a high sensitivity of 425.9 μA mM^−1^ cm^−2^.

In addition, cellulose-based function materials have been widely used in supercapacitors [140] and sensors [141]. As biomimetic materials, cellulose-based function materials also are used in sweat glucose sensors. For example, Ramadoss et al. [128] reported glucose oxidase-free polymer materials made of polyelectrolytic cellulose derivatives and an organic polycarboxylic acid for glucose sensing properties by electrochemical analysis.

### 3.2. Biomimetic Nanomaterials for Tear Glucose Sensors

Compared with sweat glucose, glucose in tears is well maintained and relatively stable [142]. Glucose content range in tears is about 0–3.6 mM for a healthy human and will be up to 4.7 mM for diabetics [143]. Tears are easy to collect and glucose content in tears can properly reflect the physiological information and disease characteristics [144]. The method of using tears can meet the requirements of sensitivity, ease of use, low cost, and minimal sampling and measurement steps for glucose sensors. Based on the above point of view, the use of a glucose sensor to detect glucose in human tears to indirectly assess blood glucose level is a reasonable and feasible solution. In addition, flexible sensors combined with wireless devices can be integrated into contact lenses for direct tear glucose detection, which are portable and comfortable. This strategy will provide a basis for continuous noninvasive glucose monitoring and is important for wearable detection devices.

**Figure 12:**
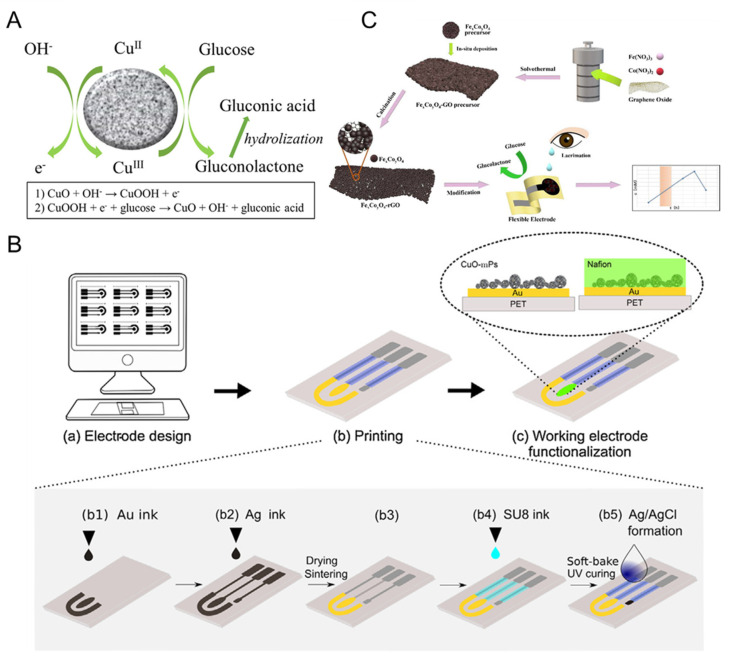
(**A**) CuO electro-oxidation mechanism of glucose in alkaline solutions; (**B**) scheme illustration of inkjet-printed electrochemical sensor based on CuO nanoparticles [106] with permission (Copyright © 2018, Elsevier); (**C**) scheme illustration of the preparation of Fe_x_Co_y_O_4_-rGO and the analysis of tear glucose [108] with permission (Copyright © 2023, Elsevier).

Early reported biomimetic nanomaterials for electrochemical tear glucose sensors are CuO nanoparticles. The mechanism for CuO sensing glucose in alkaline solution relies on the conversion of CuO into oxidizing Cu(III) species, such as CuOOH (CuO + OH^−^ → CuOOH) or Cu(OH)_4_^−^ (CuO + H_2_O + 2OH^−^ → Cu(OH)_4_^−^ + e^−^). Such Cu(III) species oxidize glucose into gluconolactone (Cu(III) + glucose → gluconolactone + Cu(II)), which will be converted into gluconic acid (Gluconolactone → gluconicacid) by hydrolyzation (Figure 12A) [106,145,146]. Using CuO nanoparticles as biomimetic nanomaterials, Romeo et al. [106] presented a flexible inkjet-printed electrochemical sensor by drop-casting CuO NWs on a Au electrode supported by polyethylene terephthalate (PET) substrate for non-enzyme tear glucose detection, as seen in Figure 12B. This sensor exhibited a detection limit of 2.99 μM, with a linear content range of 3–80 μM for glucose electrochemical sensing, and the sensor was applied in detecting tear glucose. To further enhance the sensing properties, Kheirabadi et al. [107] fabricated the screen-printed carbon electrode (SPCE) using multi-walled carbon nanotubes decorated with copper (II) oxide (MWCNT/CuO) as the biomimetic nanomaterial for tear glucose sensing. An MWCNT/CuO-based tear glucose sensor showed a lower detection limit of 1.7 μM, with a wide linear response from 5.0 to 620.0 μM.

Recently, Fe/Co bimetallic oxides were also assembled in electrochemical sensor device for tear glucose detection. As seen in Figure 12C, Zhou et al. [108] deposited Fe_x_Co_y_O_4_ on reduced graphene oxide (Fe_x_Co_y_O_4_-rGO) through simple hydrothermal reaction and calcination. The flexible electrode modified by Fe_x_Co_y_O_4_-rGO exhibited a low detection limit of 0.07 μM and a high sensitivity of 1510 μM cm^−2^ mA^−1^. The constructed Fe_x_Co_y_O_4_-rGO-based flexible sensor was used in the dynamic measurement of glucose content in tears and the results were the same as the analysis of a commercial test kit, indicating its application prospects in noninvasive diabetes diagnosis. Nevertheless, further studies are needed on human tears using analytical platforms for future practical applications.

### 3.3. Biomimetic Nanomaterials for Urine Glucose Sensors

Urine is the waste product of human metabolism, consisting of several analytes, such as urea, uric acid, and creatinine. It gives a basis for monitoring an overall physical health condition [147]. Normally, the urine measurement is positive when the glucose concentration is over 2.8 mM [148]. Materials modified with GDH have been assembled on paper-based biofuel cells using urine glucose as fuel, which reduced the burden of nursing care [149].

**Figure 13:**
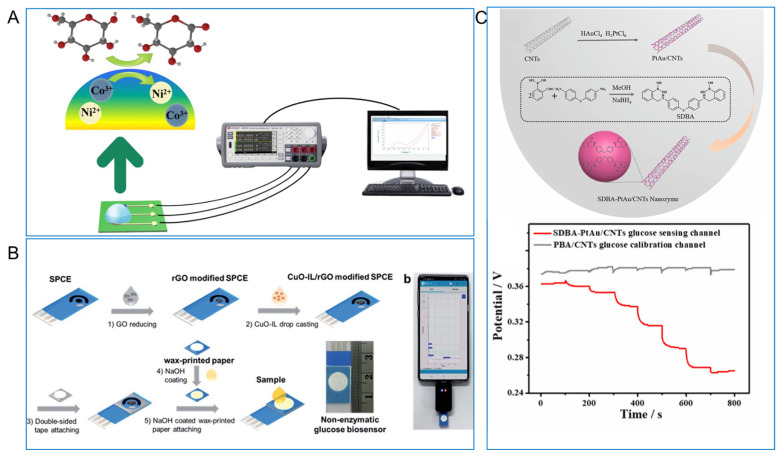
(**A**) NiCo_2_O_4_ nanosphere based glucose detection platform for urine glucose sensor [109] with permission (Copyright © 2021, IEEE); (**B**) fabrication of CuO-IL/rGO modified SPCE for nonenzymatic urine glucose sensor [110] with permission (Copyright © 2021, The Royal Society of Chemistry); (**C**) illustration of SDBA-PtAu/CNTs nanozyme and glucose standard solution at different concentrations of SDBA-PtAu/CNTs and PBA/CNTs [11] with permission (Copyright © 2023, Elsevier).

As nonenzymatic sensing materials, different biomimetic nanomaterials have been used in electrochemical urine glucose sensors. For example, Chen et al. [109] fabricated a NiCo_2_O_4_ spheres-based glucose measurement system, as seen in Figure 13A. Firstly, NiCo_2_O_4_ nanospheres lose electrons and are oxidized to NiOOH and CoOOH in the alkaline electrolyte (NiCO_2_O_4_ + OH^−^ + H_2_O → NiOOH + 2CoOOH + e^−^). Then, glucose will dissociate and convert into gluconolactone by releasing two electrons on the surface of the electrode, while NiOOH and CoOOH receive electrons and change into Ni(OH)_2_ and Co(OH)_2_ (NiOOH + Glucose → Ni(OH)_2_ + Gluconolactone; CoOOH + Glucose → Co(OH)_2_ + Gluconolactone) [109,150]. As seen in Figure 13B, Janmee et al. [110] reported nanocomposite made of copper oxide nanoparticles, ionic liquid, and reduced graphene oxide (CuO-IL/rGO) as active sensing materials and modified CuO-IL/rGO on the SPCE to detect glucose in urine. Besides metal oxides, some noble metal nanoparticles were also used to detect urine glucose. Wang et al. [11] took the detection of glucose in urine as a model to study the reliability of sensors using PtAu/CNT. As seen in Figure 13C, PtAu/CNT was modified with a PtAu/CNTs nanozyme modified by ((((thiobis(4,1-phenylene))bis(azanediyl))bis(methylene))bis(2,1phenylene))diboronic acid molecules and showed a highly improved selectivity for glucose than phenylboronic acid (PBA).

### 3.4. Biomimetic Nanomaterials for Saliva Glucose Sensors

**Figure 14:**
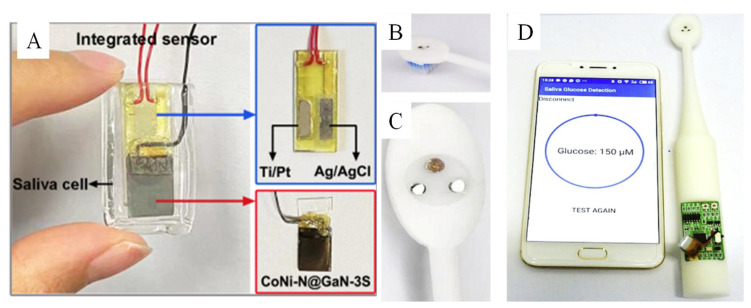
(**A**) The integrated saliva glucose sensor based on CoNi-N@GaN-3S [102] with permission (Copyright © 2022, American Chemical Society); (**B**–**D**) photograph of the smart toothbrush with electrochemical three electrodes for detecting saliva glucose and linked with APP by a smartphone [119] (Copyright © 2019, Elsevier).

Saliva consists of many biomarkers, such as lactate, glucose, phosphate, antibodies, and so on [37]. Because of easy sample collection and good correlation with blood glucose, saliva is an ideal human body fluid for noninvasive glucose sensing [151,152]. The normal level of saliva glucose in healthy individuals is 0.23–0.38 mM and, in a diabetic patient, the level range is 0.55–1.77 mM [152,153,154]. We summarize the biomimetic nanomaterials used to detect the saliva glucose by electrochemical method, as seen in Table 1. It was found that biomimetic nanomaterials were focused on detecting glucose in saliva by adding saliva into alkaline electrolyte at an early stage. Recently, researchers paid attention to the direct detection of glucose in saliva by devices made of biomimetic nanomaterials. For example, Chen et al. [102] fabricated a CoNi-N nanosheet-coated GaN 3D scaffold (CoNi-N@GaN-3S) and assembled it with Ti/Pt and Ag/AgCl as the integrated sensor for detecting saliva glucose directly (Figure 14A). Furthermore, bronze as the active sensing biomimetic nanomaterial was integrated into a smart toothbrush for monitoring of saliva glucose directly, as seen in Figure 14B [102]. This smart toothbrush is potentially a useful product for noninvasive screening of diabetes in the future.

Although various biomimetic nanomaterials have been studied to invest their glucose sensing performance, the unique biomimetic nanomaterials which are suitable for electrochemical noninvasive devices are not abundant. Therefore, there is a need to design and develop more wearable noninvasive biomimetic nanomaterials for use in glucose sensors. In addition, there is an urgent need to develop devices that can meet daily needs and be mass produced.

Over the past few decades, many biomimetic nanomaterials have been investigated for glucose-free sensing. However, there are some problems that need attention. In our evaluation, few articles have published and further elaborated the catalytic mechanism of the biomimetic materials used, which should be further investigated in future work. In addition, although these materials offer some advantages over traditional enzyme-based sensors, there are still some challenges to be addressed, such as limited understanding of their behavior and properties, their long-term stable performance, and potential side effects. In addition, integrating them into sensing devices can be challenging and require specialized manufacturing techniques and equipment, which may affect their marketability and increase costs.

## 4. Conclusions and Future Direction

Biomimetic nanomaterials play an important role in the construction and application of glucose sensors because of their unique catalytic activity, selectivity, and especially stability. In this review, the methods to enhance the glucose sensing performance of biomimetic nanomaterials containing noble-metal-based materials, transition-metal-based materials, and carbon-based materials have been discussed. In addition, we summarized the biomimetic nanomaterials used in noninvasive electrochemical glucose sensors and introduced the corresponding glucose devices for sweat, tears, saliva, and urine.

In the future, the prospect of biomimetic nanomaterials may undergo continued research into new materials with large surface areas, active sites, and co-reactive groups (binding and catalytic groups) to achieve more efficient and stable systems. In view of the problems, the author gives the following suggestions: (1) rational design of nano-enzyme materials to understand the catalytic mechanism of nano-enzyme and clarify the relationship between the structure of biomimetic nanomaterials and catalytic activity. The defects of nanomesas can be avoided through rational design of materials to enhance their sensing properties. (2) Regulate the size of biomimetic nanomaterials to improve their catalytic performance. The regulatory mechanism and means of the above-mentioned materials can also be used for reference to other biomimetic nanomaterials and their electrochemical glucose sensors. In practical application, there is still a lack of relevant technology and products in the market. It is expected that the combination of biomimetic nanomaterials and a variety of mature technologies in the market can effectively promote their early application and marketing.

## Figures and Tables

**Table 1:** A summary of biomimetic nanomaterials.

Biomimetic Nanomaterials	LOD (μM)	Sensitivity (μA mM^−1^ cm^−2^)	Linear Range (mM)	Electrolyte	Reference
C-doped Co_3_O_4_	3.86	/	0.1–0.6	/	[32]
Au/MXene	200	22.45	1–12	0.1 M NaOH	[55]
Au@Ni-Fe PBA	4.686	8.037	0.010–16	0.1 M NaOH	[56]
Pt/HCS	100	/	0.3–10 10–50	0.1 M PBS (pH 7.4)	[57]
Fe_3_O_4_@Au@CoFe-LDH	12.7	6342	0.0375–15.64	1.0 M KOH	[58]
Au_200_Bi	8.7	664	0.013–3.3	0.1 M NaOH	[61]
Au/Cu_2_O/AZO NRs Ag/Cu_2_O/AZO NRs	/	186.3 221.6	2.22–11.11 3.33–11.11	0.1 M NaOH	[62]
CuO-TiO_2_	0.6	244.2	/	0.1 M KOH	[67]
Fe-doped NiCo_2_O_4_	0.19	3055.7	0.0002–3.1	0.1 M NaOH	[68]
Cu_3_Pt/Cu_2_O	/	5082 3331 118	0.005–0.1 0.1–1.5 1.5–10	0.5 M NaOH	[69]
Pd-Co_3_O_4_	10	/	1–6.0	0.1 M NaOH	[70]
Pd-doped NiCo_2_O_4_	0.28	40.03	0.005–0.09	0.1 M NaOH	[71]
5%TiO_2_/Cu_2_O/CuO/ITO	0.25	2074.7 1099.1	0–2 2–5	0.1 M NaOH	[72]
N-O_v_/NiCo_2_O_4_-350	0.02	29,811.53	0–555	1.0 M KOH	[73]
CuCo_2_O_4_@NiCo_2_O_4_	0.35	11.12	0.001–1.158	0.1 M NaOH	[74]
NiCo_2_O_4_	86	779.35	0.0001–15.28	0.5 M NaOH	[75]
Co_3_O_4_/NiCo_2_O_4_/CC	0.64	12.835	0.001–1.127	0.1 M NaOH	[76]
NiCo_2_O_4_ HNCs	0.027	1306	0.000180–5.1	0.1 M NaOH	[77]
CuO@CNTFs	1.4	3000	0–13	0.1 M KOH	[83]
MWCNT/ZnO QDs	0.208	/	0.0001–0.002.5	0.1 M PBS (pH 7.0)	[84]
Cu, N-CQDs	1.22	/	5–700	0.1 M NaOH	[85]
CQDs/Cu_2_O	6	2900 ± 200	0.0013–6	0.1 M NaOH	[86]
NH_2_-GQDs/NiCo_2_O_4_	0.27	1185.58 2521.33	0.001–0.159 0.159–0.949	0.1 M NaOH	[88]
Au@GQDs	9.12	/	/	0.1 M NaOH	[89]
PtNi	16	24.03	0.5–15	0.1 M PBS (pH 7.4)	[91]
Ni/NiO	0.032	3251.8	0.001–3.568	0.1 M NaOH	[92]
Cu(II)/rGO	65	172	0.10–12.5	0.1 M NaOH	[96]

**Table 2:** Biomimetic nanomaterials for noninvasive glucose sensors.

Biomimetic Nanomaterials	Electrolyte	Potential (V)	LOD (μM)	Linear Range (μM)	References
Ni-Co MOF/Au/PDMS	Human sweat	0.55	/	20–790	[7]
SWCNT/rGO/CoPc	NaOH (adding saliva)	0.55	0.12	0.3–500 500–5000	[10]
PtAu/CNT	M PBS (pH 6.1)	/	/	900–40,000	[11]
Pt/MXene	PBS (pH 7.4) (adding sweat)	0.1	/	0–8000	[103]
Cu_x_O NFs	Human sweat	0.55	0.0791	>2500	[104]
Ni-Co MOF/Ag/rGO/PU	Human sweat	0.5	/	10–660	[105]
MWCNT/CuO	NaOH (adding tears)	0.4	1.7	5–620	[106]
CuO-µPs	NaOH (adding tears)	0.4	2.99	3–700	[107]
Fe_x_Co_y_O_4_-rGO	Human tears	0.55	0.07	0.1–906.4 906.4–1906.4	[108]
NiCo_2_O_4_	Human urine	1.16	0.376	1–100,000	[109]
CuO-IL/rGO	PBS(adding urine)	0.4	190	30–7000	[110]
Co_3_O_4_	NaOH (adding saliva)	0.535	0.0093	0–3000	[111]
Au/Co_3_O_4_	Synthetic saliva	0.1	20	20–1000	[112]
AuNP/Chitosan/CeO_2_	NaOH (adding saliva)	0.2	2.86	20–600	[113]
Au/CuO	NaOH (adding saliva)	0.55	0.17	5–1325	[114]
porous CuO	NaOH (adding saliva)	0.6	0.41	5–225	[115]
CuO nanoarray	NaOH (adding saliva)	0.55	0.1	1–6000	[116]
NiO	NaOH (adding saliva)	/	0.084	5–825	[117]
IrO_2_@NiO	NaOH (adding saliva)	0.35	5	0–180	[118]
CoNi-N@GaN-3S	Human saliva	0.55	5	5–1000	[102]
Bronze	Human saliva	0.65	4.7	20–320	[119]

## References

[1] Malena L., Fiser O., Stauffer P.R., Drizdal T., Vrba J., Vrba D. (2021). Feasibility evaluation of metamaterial microwave sensors for non-invasive blood glucose monitoring. Sensors.

[2] Gilnezhad J., Firoozbakhtian A., Hosseini M., Adel S., Xu G., Ganjali M.R. (2023). An enzyme-free Ti_3_C_2_/Ni/Sm-LDH-based screen-printed-electrode for real-time sweat detection of glucose. Anal. Chim. Acta.

[3] International Diabetes Federation (2021). IDF Diabetes Atlas.

[4] Wilkinson E. (2022). World health assembly ratifies first global diabetes targets. Lancet Diabetes Endocrinol..

[5] Naderi L., Shahrokhian S., Amini M.K., Hafezi Kahnamouei M. (2023). Comparison of electrocatalytic performance of CuCo_2_O_4_ nanorods and nanospheres decorated with Co_3_S_4_ nanosheets for electrochemical sensing of hydrogen peroxide and glucose in human serum. ACS Appl. Nano Mater..

[6] Zhao Y., Zhai Q., Dong D., An T., Gong S., Shi Q., Cheng W. (2019). Highly stretchable and strain-insensitive fiber-based wearable electrochemical biosensor to monitor glucose in the sweat. Anal. Chem..

[7] Shu Y., Shang Z., Su T., Zhang S., Lu Q., Xu Q., Hu X. (2022). A highly flexible Ni-Co MOF nanosheet coated Au/PDMS film based wearable electrochemical sensor for continuous human sweat glucose monitoring. Analyst.

[8] Zhu Y., Qi Y., Xu M., Luo J. (2023). Flexible biosensor based on signal amplification of gold nanoparticles-composite flower clusters for glucose detection in sweat. Colloids Surf. A Physicochem. Eng. Asp..

[9] Yang X., Yao H., Zhao G., Ameer G.A., Sun W., Yang J., Mi S. (2020). Flexible, wearable microfluidic contact lens with capillary networks for tear diagnostics. J. Mater. Sci..

[10] Adeniyi O., Nwahara N., Mwanza D., Nyokong T., Mashazi P. (2021). Nanohybrid electrocatalyst based on cobalt phthalocyanine-carbon nanotube-reduced graphene oxide for ultrasensitive detection of glucose in human saliva. Sens. Actuators B Chem..

[11] Wang F., Zhang J., Zhang M., Xu C., Cheng S., Wang Q., Zhang F., He X., He P. (2023). A multi-calibration potentiometric sensing array based on diboronic acid-PtAu/CNTs nanozyme for home monitoring of urine glucose. Anal. Chim. Acta.

[12] Wei M., Li X., Serpe M.J. (2019). Stimuli-responsive microgel-based surface plasmon resonance transducer for glucose detection using a competitive assay with concanavalin a. ACS Appl. Polym. Mater..

[13] Liu X., Huang D., Lai C., Qin L., Zeng G., Xu P., Li B., Yi H., Zhang M. (2019). Peroxidase-like activity of smart nanomaterials and their advanced application in colorimetric glucose biosensors. Small.

[14] Yue J.Y., Ding X.L., Wang L., Yang R., Bi J.S., Song Y.W., Yang P., Ma Y., Tang B. (2021). Novel enzyme-functionalized covalent organic frameworks for the colorimetric sensing of glucose in body fluids and drinks. Mater. Chem. Front..

[15] Tong L., Wu L., Zai Y., Zhang Y., Su E., Gu N. (2022). Paper-based colorimetric glucose sensor using prussian blue nanoparticles as mimic peroxidase. Biosens. Bioelectron..

[16] Naveen Prasad S., Anderson S.R., Joglekar M.V., Hardikar A.A., Bansal V., Ramanathan R. (2022). Bimetallic nanozyme mediated urine glucose monitoring through discriminant analysis of colorimetric signal. Biosens. Bioelectron..

[17] Kawin K., Frank John R., Sameer S. (2023). Nanomaterials integrated with microfluidic paper-based analytical devices for enzyme-free glucose quantification. Talanta.

[18] Jin H., Lee W.H., Cho Y.H., Han J., Im C., Yu S., Li L., Lee J., Yin Z., Kim Y.S. (2022). Self-powered illuminating glucose sensor. Nano Energy.

[19] Davies S., Hu Y., Blyth J., Jiang N., Yetisen A.K. (2023). Reusable dual-photopolymerized holographic glucose sensors. Adv. Funct. Mater..

[20] Jia Y., Zhao S., Qu Q., Yang L. (2022). Nano-channel confined biomimetic nanozyme/bioenzyme cascade reaction for long-lasting and intensive chemiluminescence. Biosens. Bioelectron..

[21] Lu J., Wang Y., Shan X., Sun Z., Zhang X., Zhao Y., Hu Y., Sun E., Tian L. (2021). Synergistic enhancement effects of cobalt oxide doped silver oxide and porphyrin zinc on an electrochemiluminescence sensor for detection of glucose. Microchem. J..

[22] Li J.X., Zhang W.H., Tong Z.R., Liu J.W. (2021). Fiber optic sensor modified by graphene oxide–glucose oxidase for glucose detection. Opt. Commun..

[23] Soundaram Jeevarathinam A., Saleem W., Martin N., Hu C., McShane M.J. (2023). NIR luminescent oxygen-sensing nanoparticles for continuous glucose and lactate monitoring. Biosensors.

[24] Rahsepar M., Foroughi F., Kim H. (2019). A new enzyme-free biosensor based on nitrogen-doped graphene with high sensing performance for electrochemical detection of glucose at biological pH value. Sens. Actuators B Chem..

[25] Lee H., Hong Y.J., Baik S., Hyeon T., Kim D.H. (2018). Enzyme-based glucose sensor: From invasive to wearable device. Adv. Health Mater..

[26] Updike S.J., Hicks G.P. (1967). The enzyme electrode. Nature.

[27] Tseng T.F., Yang Y.L., Lin Y.J., Lou S.L. (2010). Effects of electric potential treatment of a chromium hexacyanoferrate modified biosensor based on PQQ-dependent glucose dehydrogenase. Sensors.

[28] Chavez-Urbiola I.R., Reséndiz-Jaramillo A.Y., Willars-Rodriguez F.J., Martinez-Saucedo G., Arriaga L.G., Jesús A.-P., Ricardo A.E.V., Ledesma-García J. (2022). Glucose biosensor based on a flexible Au/ZnO film to enhance the glucose oxidase catalytic response. J. Electroanal. Chem..

[29] Sakalauskiene L., Popov A., Kausaite-Minkstimiene A., Ramanavicius A., Ramanaviciene A. (2022). The impact of glucose oxidase immobilization on dendritic gold nanostructures on the performance of glucose biosensors. Biosensors.

[30] Aida A., Morteza H., Ehsan S., Mohammad Reza G. (2022). Peroxidase effect of Ce_2_(WO_4_)_3_ nanoparticles to detection of glucose as a colorimetric sensor. ChemistrySelect.

[31] Stasyuk N., Smutok O., Demkiv O., Prokopiv T., Gayda G., Nisnevitch M., Gonchar M. (2020). Synthesis, catalytic properties and application in biosensorics of nanozymes and electronanocatalysts: A review. Sensors.

[32] Kang Z.W., Zhang J.Y., Li Z.Z., Kankala R.K., Wang S.B., Chen A.Z. (2023). Supercritical fluid-assisted fabrication of C-doped Co_3_O_4_ nanoparticles based on polymer-coated metal salt nanoreactors for efficient enzyme-mimicking and glucose sensor properties. Nano Res..

[33] He C., Asif M., Liu Q., Xiao F., Liu H., Xia B.Y. (2022). Noble metal construction for electrochemical nonenzymatic glucose detection. Adv. Mater. Technol..

[34] Shen M., Li W., Chen L., Chen Y., Ren S., Han D. (2021). NiCo-LDH nanoflake arrays-supported au nanoparticles on copper foam as a highly sensitive electrochemical non-enzymatic glucose sensor. Anal. Chim. Acta.

[35] Tran H.V., Nguyen N.D., Tran C.T.Q., Tran L.T., Le T.D., Tran H.T.T., Piro B., Huynh C.D., Nguyen T.N., Nguyen N.T.T. (2020). Silver nanoparticles-decorated reduced graphene oxide: A novel peroxidase-like activity nanomaterial for development of a colorimetric glucose biosensor. Arab. J. Chem..

[36] Zhao W., Zhang G., Du Y., Chen S., Fu Y., Xu F., Xiao X., Jiang W., Ji Q. (2021). Sensitive colorimetric glucose sensor by iron-based nanozymes with controllable Fe valence. J. Mater. Chem. B.

[37] Zhu B., Li X., Zhou L., Su B. (2021). An overview of wearable and implantable electrochemical glucose sensors. Electroanalysis.

[38] Peng Z., Xie X., Tan Q., Kang H., Cui J., Zhang X., Li W., Feng G. (2022). Blood glucose sensors and recent advances: A review. J. Innov. Opt. Health Sci..

[39] Zhang C., Zhang Z., Yang Q., Chen W. (2018). Graphene-based electrochemical glucose sensors: Fabrication and sensing properties. Electroanalysis.

[40] Wei M., Qiao Y., Zhao H., Liang J., Li T., Luo Y., Lu S., Shi X., Lu W., Sun X. (2020). Electrochemical non-enzymatic glucose sensors: Recent progress and perspectives. Chem. Commun..

[41] Akter R., Saha P., Shah S.S., Shaikh M.N., Aziz M.A., Ahammad A.J.S. (2022). Nanostructured nickel-based non-enzymatic electrochemical glucose sensors. Chem. Asian J..

[42] Radhakrishnan S., Lakshmy S., Santhosh S., Kalarikkal N., Chakraborty B., Rout C.S. (2022). Recent developments and future perspective on electrochemical glucose sensors based on 2D materials. Biosensors.

[43] Mohamad Nor N., Ridhuan N.S., Abdul Razak K. (2022). Progress of enzymatic and non-enzymatic electrochemical glucose biosensor based on nanomaterial-modified electrode. Biosensors.

[44] Li J.Y., Zhu J., Weng G.J., Li J.J., Zhao J.W. (2022). Multiplex sensing based on plasmonic optics of noble metallic nanostructures. Crit. Rev. Anal. Chem..

[45] Takashima T., Fujishiro Y., Irie H. (2020). Noble metal modification of CdS-covered CuInS_2_ electrodes for improved photoelectrochemical activity and stability. Catalysts.

[46] Chen J., Liu X., Zheng G., Feng W., Wang P., Gao J., Liu J., Wang M., Wang Q. (2023). Detection of glucose based on noble metal nanozymes: Mechanism, activity regulation, and enantioselective recognition. Small.

[47] Chang L., Shuaishuai Z., Fei G., Jianhan L., Juewen L., Jinkai Z. (2022). DNA-mediated growth of noble metal nanomaterials for biosensing applications. Trends Anal. Chem..

[48] Yang T.H., Ahn J., Shi S., Wang P., Gao R., Qin D. (2021). Noble-metal nanoframes and their catalytic applications. Chem. Rev..

[49] Azharuddin M., Zhu G.H., Das D., Ozgur E., Uzun L., Turner A.P.F., Patra H.K. (2019). A repertoire of biomedical applications of noble metal nanoparticles. Chem. Commun..

[50] Toumey C. (2007). The man who understood the Feynman machine. Nat. Nanotechnol..

[51] Trung B.C., Tu L.N.Q., Thanh L.D., Van Dung N., An N.T., Long N.Q. (2020). Combined adsorption and catalytic oxidation for low-temperature toluene removal using nano-sized noble metal supported on ceria-granular carbon. J. Environ. Chem. Eng..

[52] Tang Z., Zhang Y., Deng X., Dai Y., Zhang W., Fan F., Qing B., Zhu C., Fan J., Shi Y. (2018). The H_2_ sensing properties of facets-dependent Pd nanoparticles-supported ZnO nanorods. Dalton Trans..

[53] Longato A., Vanzan M., Colusso E., Corni S., Martucci A. (2023). Enhancing tungsten oxide gasochromism with noble metal nanoparticles: The importance of the interface. Small.

[54] Tong Y., Xue G., Wang H., Liu M., Wang J., Hao C., Zhang X., Wang D., Shi X., Liu W. (2018). Interfacial coupling between noble metal nanoparticles and metal–organic frameworks for enhanced catalytic activity. Nanoscale.

[55] Bi C., Song S.X., Li H.S., Peng H.L., Li Q.F. (2022). Non-enzymatic glucose sensor based on porous foam Au/Mxene nanocomposites. Chin. J. Chem. Phys..

[56] Shen L., Liang Z., Chen Z., Wu C., Hu X., Zhang J., Jiang Q., Wang Y. (2022). Reusable electrochemical non-enzymatic glucose sensors based on Au-inlaid nanocages. Nano Res..

[57] Zhang C., Zhang R., Gao X., Cheng C., Hou L., Li X., Chen W. (2018). Small naked Pt nanoparticles confined in mesoporous shell of hollow carbon spheres for high-performance nonenzymatic sensing of H_2_O_2_ and glucose. ACS Omega.

[58] Sun F., Wang X., You Z., Xia H., Wang S., Jia C., Zhou Y., Zhang J. (2022). Sandwich structure confined gold as highly sensitive and stable electrochemical non-enzymatic glucose sensor with low oxidation potential. J. Mater. Sci. Technol..

[59] Zhong S.L., Zhuang J., Yang D.P., Tang D. (2017). Eggshell membrane-templated synthesis of 3D hierarchical porous Au networks for electrochemical nonenzymatic glucose sensor. Biosens. Bioelectron..

[60] Hsu C.L., Fang Y.J., Hsueh T.J., Wang S.H., Chang S.J. (2017). Nonenzymatic glucose sensor based on Au/ZnO core–shell nanostructures decorated with au nanoparticles and enhanced with blue and green light. J. Phys. Chem. B.

[61] Fang Q., Qin Y., Wang H., Xu W., Yan H., Jiao L., Wei X., Li J., Luo X., Liu M. (2022). Ultra-low content bismuth-anchored gold aerogels with plasmon property for enhanced nonenzymatic electrochemical glucose sensing. Anal. Chem..

[62] Chen H.C., Yeh Y.C., Yen M.H. (2022). Synthesis of Au or Ag/Cu_2_O/aluminum doped zinc oxide nanorods hybrid electrode for high sensitive non-enzymatic glucose sensor: Mechanism investigation of formation and surface plasmon resonance. Mater. Chem. Phys..

[63] Weiran Z., Yong L., Lawrence Yoon Suk L. (2022). Bismuth and metal-doped bismuth nanoparticles produced by laser ablation for electrochemical glucose sensing. Sens. Actuators B Chem..

[64] Niu X., Li X., Pan J., He Y., Qiu F., Yan Y. (2016). Recent advances in non-enzymatic electrochemical glucose sensors based on non-precious transition metal materials: Opportunities and challenges. RSC Adv..

[65] Meng C., Ling T., Ma T.Y., Wang H., Hu Z., Zhou Y., Mao J., Du X.W., Jaroniec M., Qiao S.Z. (2017). Atomically and electronically coupled Pt and CoO hybrid nanocatalysts for enhanced electrocatalytic performance. Adv. Mater..

[66] Lu H., Li X., Monny S.A., Wang Z., Wang L. (2022). Photoelectrocatalytic hydrogen peroxide production based on transition-metal-oxide semiconductors. Chin. J. Catal..

[67] Tobaldi D.M., Espro C., Leonardi S.G., Lajaunie L., Seabra M.P., Calvino J.J., Marini S., Labrincha J.A., Neri G. (2020). Photo-electrochemical properties of CuO–TiO_2_ heterojunctions for glucose sensing. J. Mater. Chem. C.

[68] Qi C., Zhang C., Yang Z. (2022). Fe doping induced formation of crystalline/amorphous NiCo_2_O_4_ core/shell heterostructure for highly sensitive nonenzymatic detection of glucose. J. Alloys Compd..

[69] Yang B., Han N., Zhang L., Yi S., Zhang Z., Wang Y., Zhou Y., Chen D., Gao Y. (2020). Cu_3_Pt/Cu_2_O nanorod array prepared by a facile method for glucose detection. Appl. Surf. Sci..

[70] Chang A.S., Tahira A., Solangi Z.A., Solangi A.G., Ibupoto M.H., Chang F., Medany S.S., Nafady A., Kasry A., Willander M. (2022). Pd-Co_3_O_4_-based nanostructures for the development of enzyme-free glucose sensor. Bull. Mater. Sci..

[71] Naik K.K., Gangan A., Chakraborty B., Nayak S.K., Rout C.S. (2017). Enhanced nonenzymatic glucose-sensing properties of electrodeposited NiCo_2_O_4_-Pd nanosheets: Experimental and DFT investigations. ACS Appl. Mater. Interfaces.

[72] Hou S., Lu N., Zhu Y., Zhang J., Zhang X., Yan Y., Zhang P., Zhang Z. (2022). Photoinduced phase-transition on CuO electrospun nanofibers over the TiO_2_ photosensitizer for enhancing non-enzymatic glucose-sensing performance. J. Alloys Compd..

[73] Seong J., Patil A.M., Roy S.B., Lee J., Jun S.C. (2022). N-doped oxygen vacancy-rich NiCo_2_O_4_ nanoarrays for supercapacitor and non-enzymatic glucose sensing. Int. J. Energy Res..

[74] Liu S., Zeng W., Guo Q., Li Y. (2020). Facile synthesis of CuCo_2_O_4_@NiCo_2_O_4_ hybrid nanowire arrays on carbon cloth for a multicomponent non-enzymatic glucose sensor. Nanotechnology.

[75] Sivakumar M., Vivekanandan A.K., Panomsuwan G., Veeramani V., Chen S.H., Jiang Z., Maiyalagan T. (2022). Flower-like NiCo_2_O_4_ nanoflake surface covered on carbon nanolayer for high-performance electro-oxidation of non-enzymatic glucose biosensor. Mater. Today Chem..

[76] Guo Q., Zeng W., Liu S., Li Y. (2020). In situ formation of Co_3_O_4_ hollow nanocubes on carbon cloth-supported NiCo_2_O_4_ nanowires and their enhanced performance in non-enzymatic glucose sensing. Nanotechnology.

[77] Feng Y., Xiang D., Qiu Y., Li L., Li Y., Wu K., Zhu L. (2019). MOF-derived spinel NiCo_2_O_4_ hollow nanocages for the construction of non-enzymatic electrochemical glucose sensor. Electroanalysis.

[78] Jose J., Prakash P., Jeyaprabha B., Abraham R., Mathew R.M., Zacharia E.S., Thomas V., Thomas J. (2023). Principle, design, strategies, and future perspectives of heavy metal ion detection using carbon nanomaterial-based electrochemical sensors: A review. J. Iran. Chem. Soc..

[79] Hassanvand Z., Jalali F., Nazari M., Parnianchi F., Santoro C. (2020). Carbon nanodots in electrochemical sensors and biosensors: A review. ChemElectroChem.

[80] Mohammadpour-Haratbar A., Mohammadpour-Haratbar S., Zare Y., Rhee K.Y., Park S.J. (2022). A review on non-enzymatic electrochemical biosensors of glucose using carbon nanofiber nanocomposites. Biosensors.

[81] Esteves L.M., Oliveira H.A., Xing Y., Passos F.B. (2021). Cobalt supported on carbon nanotubes for methane chemical vapor deposition for the production of new carbon nanotubes. New J. Chem..

[82] Lin Y., Lu F., Tu Y., Ren Z. (2004). Glucose biosensors based on carbon nanotube nanoelectrode ensembles. Nano Lett..

[83] Sheza M., Mohsin J., Sohail N., Muhammad Adeel A., Ali H., Muhammad A., Ahmad Raza A., Arif N., Munawar I., Norah A. (2023). Carbon nanotube fiber-based flexible microelectrode for electrochemical glucose sensors. ACS Omega.

[84] Vinoth V., Subramaniyam G., Anandan S., Valdés H., Manidurai P. (2021). Non-enzymatic glucose sensor and photocurrent performance of zinc oxide quantum dots supported multi-walled carbon nanotubes. Mater. Sci. Eng. B.

[85] Wu H., Yan Y., Huang Q., Liang G., Qiu F., Ye Z., Liu D. (2020). A simple, cost-effective and selective analysis of glucose via electrochemical impedance sensing based on copper and nitrogen co-doped carbon quantum dots. New J. Chem..

[86] Maaoui H., Teodoresu F., Wang Q., Pan G.H., Addad A., Chtourou R., Szunerits S., Boukherroub R. (2016). Non-enzymatic glucose sensing using carbon quantum dots decorated with copper oxide nanoparticles. Sensors.

[87] Kipnusu W.K., Doñate-Buendía C., Fernández-Alonso M., Lancis J., Mínguez-Vega G. (2020). Nonlinear optics to glucose sensing: Multifunctional nitrogen and boron doped carbon dots with solid-state fluorescence in nanoporous silica films. Part. Part. Syst. Charact..

[88] Wu M., Zhu J., Ren Y., Yang N., Hong Y., Wang W., Huang W., Si W., Dong X. (2019). NH_2_-GQDS-doped nickel-cobalt oxide deposited on carbon cloth for nonenzymatic detection of glucose. Adv. Mater. Interfaces.

[89] de Lima L.F., de Freitas A.D., Ferreira A.L., Maciel C.C., Ferreira M., de Araujo W.R. (2022). Enzymeless glucose sensor based on disposable ecoflex®/graphite thermoplastic composite substrate modified with Au@GQDS. Sens. Actuators Rep..

[90] Asen P., Esfandiar A., Kazemi M. (2022). Nonenzymatic sweat-based glucose sensing by flower-like Au nanostructures/graphene oxide. ACS Appl. Nano Mater..

[91] Li R., Deng X., Xia L. (2020). Non-enzymatic sensor for determination of glucose based on PtNi nanoparticles decorated graphene. Sci. Rep..

[92] Wang Q., Zheng S., Li T., Wang Z. (2021). Ni/NiO multivalent system encapsulated in nitrogen-doped graphene realizing efficient activation for non-enzymatic glucose sensing. Ceram. Int..

[93] Sitko R., Turek E., Zawisza B., Malicka E., Jan T. (2013). Adsorption of divalent metal ions from aqueous solutions using graphene oxide. Dalton Trans..

[94] Dong L., Chen Z., Zhao X., Ma J., Lin S., Li M., Bao Y., Chu L., Leng K., Lu H. (2020). A non-dispersion strategy for large-scale production of ultra-high concentration graphene slurries in water. Nat. Commun..

[95] Peng W., Li H., Liu Y., Song S. (2017). A review on heavy metal ions adsorption from water by graphene oxide and its composites. J. Mol. Liq..

[96] Phetsang S., Kidkhunthod P., Chanlek N., Jakmunee J., Mungkornasawakul P., Ounnunkad K. (2021). Copper/reduced graphene oxide film modified electrode for non-enzymatic glucose sensing application. Sci. Rep..

[97] Leong K.L., Ho M.Y., Lee X.Y., Yee M.S.L. (2020). A review on the development of non-enzymatic glucose sensor based on graphene-based nanocomposites. Nano.

[98] Mohan A.M.V., Rajendran V., Mishra R.K., Jayaraman M. (2020). Recent advances and perspectives in sweat based wearable electrochemical sensors. Trends Anal. Chem..

[99] Aihara M., Kubota N., Kadowaki T. (2018). Study of the correlation between tear glucose concentrations and blood glucose concentrations. Diabetes.

[100] Elsherif M., Alam F., Salih A.E., AlQattan B., Yetisen A.K., Butt H. (2021). Wearable Bifocal Contact Lens for Continual Glucose Monitoring Integrated with Smartphone Readers. Small.

[101] Ayushman R., Anupma M., Surinder S. (2023). Design of a triple-layered plasmonic biosensor for glucose monitoring from urine sample for diabetes prevention. MAPAN.

[102] Chen S., Huang H., Sun H., Liu Q., Zhu H., Zhao J., Liu P., Yu J. (2022). Electrochemical sensor made with 3D micro-/mesoporous structures of CoNi-N/GaN for noninvasive detection of glucose. ACS Appl. Mater. Interfaces.

[103] Li Q.F., Chen X., Wang H., Liu M., Peng H.L. (2023). Pt/MXene-based flexible wearable non-enzymatic electrochemical sensor for continuous glucose detection in sweat. ACS Appl. Mater. Interfaces.

[104] Yu Z., Wu H., Xu Z., Yang Z., Lv J., Kong C. (2023). Wearable noninvasive glucose sensor based on Cu_x_O NFs/Cu NPs nanocomposites. Sensors.

[105] Shu Y., Su T., Lu Q., Shang Z., Xu Q., Hu X. (2021). Highly stretchable wearable electrochemical sensor based on Ni-Co MOF nanosheet-decorated Ag/rGO/PU fiber for continuous sweat glucose detection. Anal. Chem..

[106] Romeo A., Moya A., Leung T.S., Gabriel G., Villa R., Sánchez S. (2018). Inkjet printed flexible non-enzymatic glucose sensor for tear fluid analysis. Appl. Mater. Today.

[107] Asgari Kheirabadi Z., Rabbani M. (2022). Samiei Foroushani, M. Green fabrication of nonenzymatic glucose sensor using multi-walled carbon nanotubes decorated with copper (ii) oxide nanoparticles for tear fluid analysis. Appl. Biochem. Biotechnol..

[108] Zhou F., Zhao H., Chen K., Cao S., Shi Z., Lan M. (2023). Flexible electrochemical sensor with Fe/Co bimetallic oxides for sensitive analysis of glucose in human tears. Anal. Chim. Acta.

[109] Chen S., Zhang D., Yang Y., Song X. (2021). An electrochemical nonenzymatic microsensor modified by nickel cobaltate nanospheres for glucose sensing in urine. IEEE Sens. J..

[110] Janmee N., Preechakasedkit P., Rodthongkum N., Chailapakul O., Potiyaraj P., Ruecha N. (2021). A non-enzymatic disposable electrochemical sensor based on surface-modified screen-printed electrode cuo-il/rgo nanocomposite for a single-step determination of glucose in human urine and electrolyte drinks. Anal. Methods.

[111] Wang M., Liu F., Zhang Z., Meng E., Gong F., Li F. (2020). Co_3_O_4_ nanoparticles as a noninvasive electrochemical sensor for glucose detection in saliva. Nano.

[112] Coyle V.E., Kandjani A.E., Field M.R., Hartley P., Chen M., Sabri Y.M., Bhargava S.K. (2019). Co_3_O_4_ needles on Au honeycomb as a non-invasive electrochemical biosensor for glucose in saliva. Biosens. Bioelectron..

[113] Jiang L., Xue Q., Jiao C., Liu H., Zhou Y., Ma H., Yang Q. (2018). A non-enzymatic nanoceria electrode for non-invasive glucose monitoring. Anal. Methods.

[114] Chakraborty P., Dhar S., Debnath K., Majumder T., Mondal S.P. (2019). Non-enzymatic and non-invasive glucose detection using au nanoparticle decorated CuO nanorods. Sens. Actuators B Chem..

[115] Chakraborty P., Dhar S., Deka N., Debnath K., Mondal S.P. (2020). Non-enzymatic salivary glucose detection using porous CuO nanostructures. Sens. Actuators B Chem..

[116] Yang J., Chen H., Zhu C., Huang Z., Ou R., Gao S., Yang Z. (2022). A miniature CuO nanoarray sensor for noninvasive detection of trace salivary glucose. Anal. Biochem..

[117] Chakraborty P., Deka N., Patra D.C., Debnath K., Mondal S.P. (2021). Salivary glucose sensing using highly sensitive and selective non-enzymatic porous NiO nanostructured electrodes. Surf. Interfaces.

[118] Wang J., Xu L., Lu Y., Sheng K., Liu W., Chen C., Li Y., Dong B., Song H. (2016). Engineered IrO_2_@NiO core-shell nanowires for sensitive non-enzymatic detection of trace glucose in saliva. Anal. Chem..

[119] Chen J., Zhu X., Ju Y., Ma B., Zhao C., Liu H. (2019). Electrocatalytic oxidation of glucose on bronze for monitoring of saliva glucose using a smart toothbrush. Sens. Actuators B Chem..

[120] Martín A., Kim J., Kurniawan J.F., Sempionatto J.R., Moreto J.R., Tang G., Campbell A.S., Shin A., Lee M.Y., Liu X. (2017). Epidermal microfluidic electrochemical detection system: Enhanced sweat sampling and metabolite detection. ACS Sens..

[121] Kang B.C., Park B.S., Ha T.J. (2019). Highly sensitive wearable glucose sensor systems based on functionalized single-wall carbon nanotubes with glucose oxidase-nafion composites. Appl. Surf. Sci..

[122] Poletti F., Zanfrognini B., Favaretto L., Quintano V., Sun J., Treossi E., Melucci M., Palermo V., Zanardi C. (2021). Continuous capillary-flow sensing of glucose and lactate in sweat with an electrochemical sensor based on functionalized graphene oxide. Sens. Actuators B Chem..

[123] Lin Y., Bariya M., Nyein H.Y.Y., Kivimäki L., Uusitalo S., Jansson E., Ji W., Yuan Z., Happonen T., Liedert C. (2019). Porous enzymatic membrane for nanotextured glucose sweat sensors with high stability toward reliable noninvasive health monitoring. Adv. Funct. Mater..

[124] Wei X., Zhu M., Li J., Liu L., Yu J., Li Z., Ding B. (2021). Wearable biosensor for sensitive detection of uric acid in artificial sweat enabled by a fiber structured sensing interface. Nano Energy.

[125] Sempionatto J.R., Khorshed A.A., Ahmed A., De Loyola e Silva A.N., Barfidokht A., Yin L., Goud K.Y., Mohamed M.A., Bailey E., May J. (2020). Epidermal enzymatic biosensors for sweat vitamin C: Toward personalized nutrition. ACS Sens..

[126] Manimegala P., Mary X.A., Biji N., Shiny V.S. (2021). Dehydration measurement using sweat sensor patch and pulse sensor. J. Phys. Conf. Ser..

[127] Lin P.H., Sheu S.C., Chen C.W., Huang S.C., Li B.R. (2022). Wearable hydrogel patch with noninvasive, electrochemical glucose sensor for natural sweat detection. Talanta.

[128] Ramadoss P., Rahman M.I., Perumal A., Nallaiyan R., Basha S.H., Dakshanamoorthy A. (2020). Non-invasive, non-enzymatic, biodegradable and flexible sweat glucose sensor and its electrochemical studies. ChemistrySelect.

[129] Chen Q., Liu Y., Gu K., Yao J., Shao Z., Chen X. (2022). Silk-based electrochemical sensor for the detection of glucose in sweat. Biomacromolecules.

[130] Zheng L., Liu Y., Zhang C. (2021). A sample-to-answer, wearable cloth-based electrochemical sensor (WCECS) for point-of-care detection of glucose in sweat. Sens. Actuators B Chem..

[131] Cui Y., Duan W., Jin Y., Wo F., Xi F., Wu J. (2020). Ratiometric fluorescent nanohybrid for noninvasive and visual monitoring of sweat glucose. ACS Sens..

[132] Aziz A., Asif M., Ashraf G., Iftikhar T., Hussain W., Wang S. (2022). Environmental significance of wearable sensors based on MXene and graphene. Trends Environ. Anal. Chem..

[133] Zhao Z., Sun Y., Song J., Li Y., Xie Y., Cui H., Gong W., Hu J., Chen Y. (2021). Highly sensitive nonenzymetic glucose sensing based on multicomponent hierarchical NiCo-LDH/CCCH/CuF nanostructures. Sens. Actuators B Chem..

[134] Chen Z., Guo J., Zhou T., Zhang Y., Chen L. (2013). A novel nonenzymatic electrochemical glucose sensor modified with Ni/Al layered double hydroxide. Electrochim. Acta.

[135] Eshghi A., Kheirmand M. (2017). Graphene/Ni–Fe layered double hydroxide nano composites as advanced electrode materials for glucose electro oxidation. Int. J. Hydrogen Energy.

[136] Amin K.M., Muench F., Kunz U., Ensinger W. (2021). 3D NiCo-layered double hydroxide@Ni nanotube networks as integrated free-standing electrodes for nonenzymatic glucose sensing. J. Colloid Interface Sci..

[137] Zhang Y., He Z., Dong Q., Tang X., Yang L., Huang K., Zou Z., Jiang X., Xiong X. (2022). 3D Co_x_P@NiCo-LDH heteronanosheet array: As a high sensitivity sensor for glucose. Microchem. J..

[138] Wang L., Miao X., Qu Y., Duan C., Wang B., Yu Q., Gao J., Song D., Li Y., Yin Z. (2020). Rattle-type Au@NiCo LDH hollow core-shell nanostructures for nonenzymatic glucose sensing. J. Electroanal. Chem..

[139] Zhao Z., Huang Y., Huang Z., Mei H., Xie Y., Long D., Zhu F., Gong W. (2022). Nonenzymetic glucose sensitive device based on morchella shaped nickel-copper layered double hydroxide. Appl. Surf. Sci..

[140] Xiao J., Li H., Zhang H., He S., Zhang Q., Liu K., Jiang S., Duan G., Zhang K. (2022). Nanocellulose and its derived composite electrodes toward supercapacitors: Fabrication, properties, and challenges. J. Bioresour. Bioprod..

[141] Ma H., Cheng Z., Li X., Li B., Fu Y., Jiang J. (2023). Advances and challenges of cellulose functional materials in sensors. J. Bioresour. Bioprod..

[142] Kim S., Jeon H.J., Park S., Lee D.Y., Chung E. (2020). Tear glucose measurement by reflectance spectrum of a nanoparticle embedded contact lens. Sci. Rep..

[143] Baca J.T., Finegold D.N., Asher S.A. (2007). Tear glucose analysis for the noninvasive detection and monitoring of diabetes mellitus. Ocul. Surf..

[144] Lee W.C., Koh E.H., Kim D.H., Park S.G., Jung H.S. (2021). Plasmonic contact lens materials for glucose sensing in human tears. Sens. Actuators B Chem..

[145] Marioli J.M., Kuwana T. (1992). Electrochemical characterization of carbohydrate oxidation at copper electrodes. Electrochim. Acta.

[146] Xie Y.Q., Huber C.O. (1991). Electrocatalysis and amperometric detection using an electrode made of copper oxide and carbon paste. Anal. Chem..

[147] Bi Y., Sun M., Wang J., Zhu Z., Bai J., Emran M.Y., Kotb A., Bo X., Zhou M. (2023). Universal Fully Integrated Wearable Sensor Arrays for the Multiple Electrolyte and Metabolite Monitoring in Raw Sweat, Saliva, or Urine. Anal. Chem..

[148] Zhang Z., Chen Z., Cheng F., Zhang Y., Chen L. (2017). Highly sensitive on-site detection of glucose in human urine with naked eye based on enzymatic-like reaction mediated etching of gold nanorods. Biosens. Bioelectron..

[149] Shitanda I., Fujimura Y., Takarada T., Suzuki R., Aikawa T., Itagaki M., Tsujimura S. (2021). Self-powered diaper sensor with wireless transmitter powered by paper-based biofuel cell with urine glucose as fuel. ACS Sens..

[150] Elakkiya R., Maduraiveeran G. (2019). A three-dimensional nickel–cobalt oxide nanomaterial as an enzyme-mimetic electrocatalyst for the glucose and lactic acid oxidation reaction. New J. Chem..

[151] Moyer J., Wilson D., Finkelshtein I., Wong B., Potts R. (2012). Correlation between sweat glucose and blood glucose in subjects with diabetes. Diabetes Technol. Ther..

[152] Sreedevi, Shashikanth M.C., Shambulingappa P. (2008). Comparison of serum glucose and salivary glucose in diabetic patients. J. Indian Acad. Oral Med. Radiol..

[153] Abd-Elraheem S.E., El Saeed A.M., Mansour H.H. (2017). Salivary changes in type 2 diabetic patients. Diabetes Metab. Syndr. Clin. Res. Rev..

[154] Panchbhai A.S. (2012). Correlation of salivary glucose level with blood glucose level in diabetes mellitus. J. Oral Maxillofac. Res..

